# Molecular Recognition in Confined Space Elucidated with DNA Nanopores and Single-Molecule Force Microscopy

**DOI:** 10.1021/acs.nanolett.3c00743

**Published:** 2023-05-11

**Authors:** Saanfor Hubert Suh, Yongzheng Xing, Alexia Rottensteiner, Rong Zhu, Yoo Jin Oh, Stefan Howorka, Peter Hinterdorfer

**Affiliations:** † Department of Applied Experimental Biophysics, Johannes Kepler University Linz, Institute of Biophysics, Gruberstr. 40, 4020 Linz, Austria; ‡ Department of Chemistry, University College London, Institute of Structural and Molecular Biology, 20 Gordon Street, London WC1H OAJ, United Kingdom

**Keywords:** Molecular recognition, confinement, DNA nanotechnology, AFM, nanopore

## Abstract

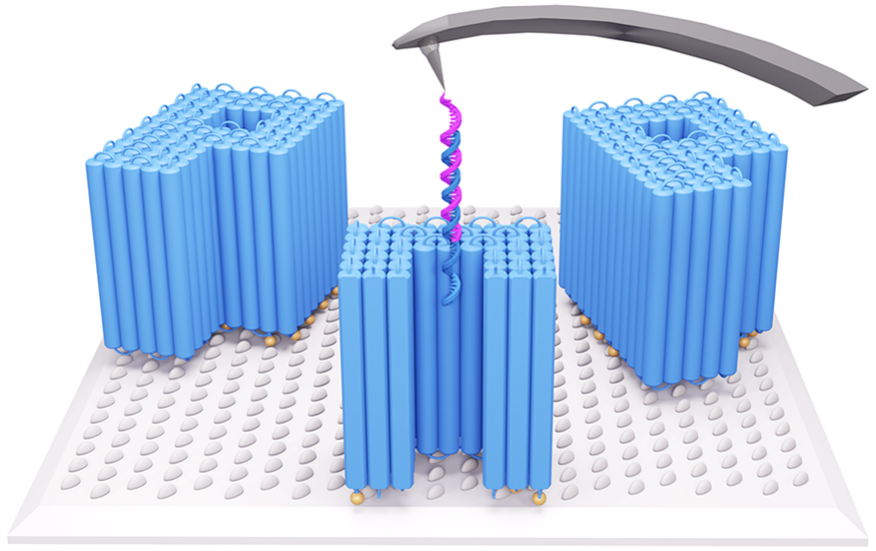

The binding of ligands to receptors within a nanoscale small space is relevant in biology, biosensing, and affinity filtration. Binding in confinement can be studied with biological systems but under the limitation that essential parameters cannot be easily controlled including receptor type and position within the confinement and its dimensions. Here we study molecular recognition with a synthetic confined nanopore with controllable pore dimension and molecular DNA receptors at different depth positions within the channel. Binding of a complementary DNA strand is studied at the single-molecule level with atomic force microscopy. Following the analysis, kinetic association rates are lower for receptors positioned deeper inside the pore lumen while dissociation is faster and requires less force. The phenomena are explained by the steric constraints on molecular interactions in confinement. Our study is the first to explore recognition in DNA nanostructures with atomic force microscopy and lays out new tools to further quantify the effect of nanoconfinement on molecular interactions.

Molecular interactions within a nanoscale confined space are relevant in several areas. In biology, membrane proteins with recessed binding sites are actuated by ligand binding, such as the serotonin receptor^1−3^ and related G-protein-coupled receptors.^4−6^ Molecular binding in nanoconfinement also occurs in several ligand-gated ion channels^7^ as well as in membrane pores that facilitate the selective transport of cargo across the hollow proteins’ channel, such as maltoporin^8^ and the nuclear pore complex.^9^ Other examples are ABC transporters that are shuttling bioactive cargo across the channel out of or into cells.^10^ In the field of biosensing, nanopores carrying engineered receptors within the channel lumen constitute the core unit for label-free sensing. The approach can detect a wide range of analytes including metal ions,^11^ small molecules,^12,13^ proteins,^14,15^ and nucleotides^16,17^ and has led to widespread DNA/RNA nanopore sequencing.^18−21^ Within filtration science, porous membranes carrying molecular binding sites at the pore walls enable affinity-based purification.

Understanding molecular interactions under nanoconfinement is fundamental for science and for rationally improving nanopore sensors and filtration membranes. Key open questions are the extent that recognition and dissociation kinetics are influenced by essential parameters. These parameters include receptor type and position, the number of receptors within the channel lumen, and the dimension of the channel. Existing studies have explored binding in confinement using a narrow protein pore,^16,22^ while theory has modeled wider synthetic channels filled with a meshwork of ligand-terminated polymers.^23−25^ However, biological membrane proteins cannot be easily engineered to freely dial-in the mentioned key parameters, while the burgeoning *de novo* design of protein nanochannels^26,27^ has not yet reached the molecular control required for tuning the parameters. Other synthetic channels made from inorganic materials are wider but cannot be easily decorated with receptors in defined number and channel position.^28,29^

Here we study molecular interaction within nanoconfinement using a synthetic nanochannel highly tunable in dimensions and receptor position (Figure 1A,B). The nanochannel is designed with DNA origami, which is a powerful route to control nanostructure dimensions^30−39^ by taking advantage of predictable base pairing.^40^ The design of our custom-made nanopore is related to previous hollow structures^41−46^ that were made to insert into bilayer membranes to control the channel flux optionally in response to external stimuli^47−52^ and for biosensing.^41,42,51,53,54^ Unlike previous structures, our current nanopore features an individual receptor for DNA strands^55−57^ at defined positions inside the pore lumen (Figure 1A). As another difference, the current DNA nanopore binds onto a solid support—in an oriented upright fashion—so that probe molecules can be threaded from the top into the pore lumen (see the Abstract graphic, magenta probe and blue receptor strand).

**Figure 1:**
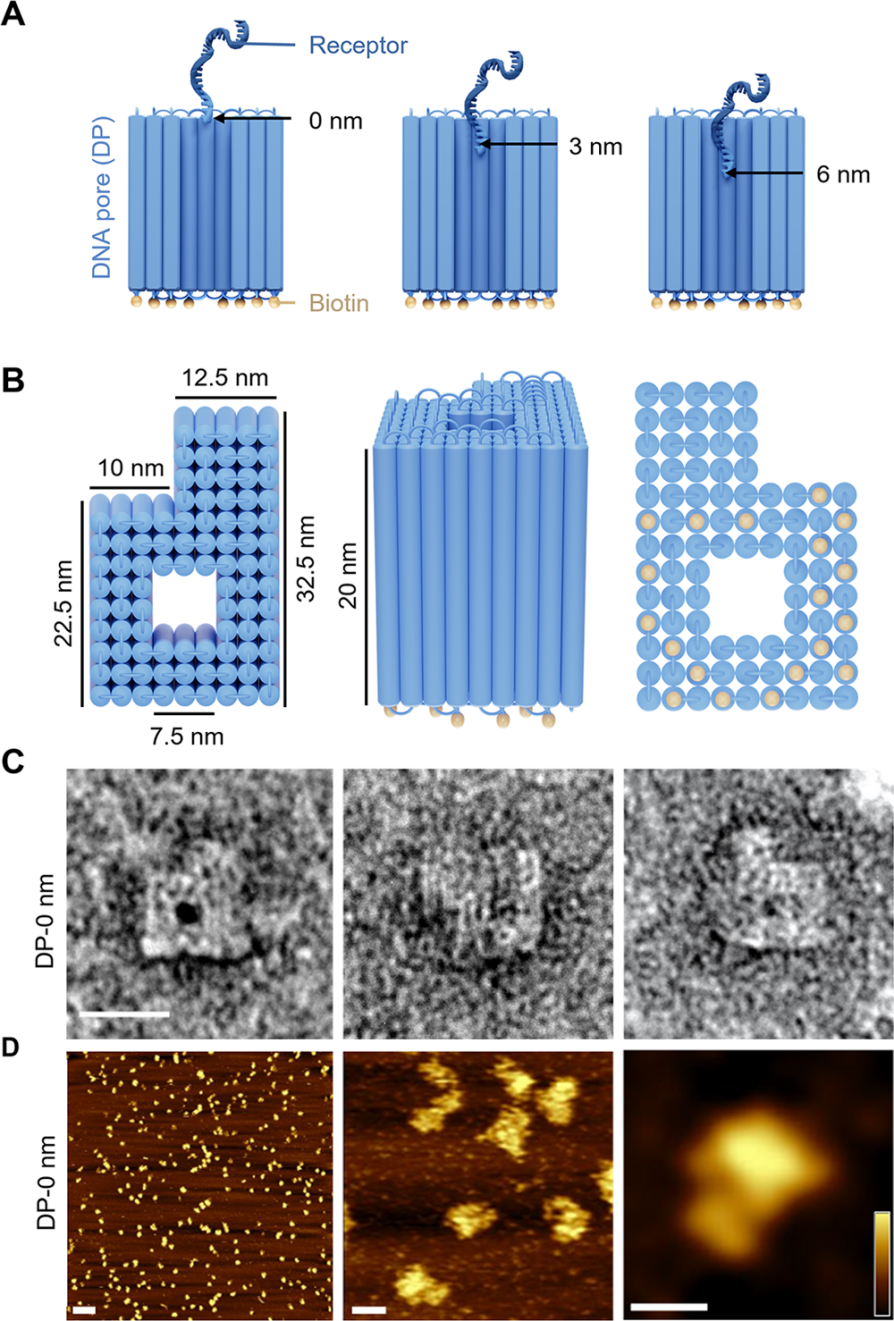
Pore design and structural characterization. (A) Cross-sectional view of a schematic model of the DNA nanopore revealing the vertically running lumen and the ssDNA receptor tethered at 0, 3, and 6 nm depth (left to right) within the pore channel relative to the pore top. DNA duplexes are illustrated as blue cylinders. (B) Schematic representation of the DNA nanopore in top, side, and bottom view, from left to right. Yellow spheres represent biotin anchors. The height of the pore is scaled up by 50% compared to the nominal dimensions for reasons of visual clarity. (C) TEM images of negatively stained DNA nanopores. Scale bar, 25 nm. (D) AFM topography images of the DNA nanopore in tapping mode after immobilization on mica. Horizontal scale bars: 250, 25, and 25 nm (left to right). Vertical scale bar: 10 nm. The vertical scale of 10 nm was chosen to enhance the contrast of the nanostructures.

To examine the interaction of the receptor in the nanoconfinement of the channel, we use the atomic force microscopy (AFM) methods^58^ of topography and recognition (TREC) imaging^59^ and single-molecule force spectroscopy (SMFS).^60^ TREC combines topography imaging with force sensing for the specific recognition and localization of biomolecules, including nanoscale targeting of DNA,^61−63^ and has also been applied for determining the position of nucleotide binding sites within membrane proteins with nanometer precision.^64^ SMFS is sufficiently sensitive to measure pico-Newton ranges of ligand/receptor dissociation forces on the single-molecule level^60^ to quantify binding strength and kinetics,^65^ as well as for mapping interaction energy landscapes^66^ and nanoscale bond mechanics.^67,68^ Recognition force microscopy has not been applied to DNA origami structures before. To maintain consistency in analysis, AFM force spectroscopy measurements were performed at DNA hybridization conditions similar to the solution-based measurements.

We designed a DNA nanopore, termed DNA nanoshell pore (DP), featuring a single ssDNA receptor at multiple sites within the pore’s channel lumen (Figure 1A). The DNA pore is composed of 92 duplexes interconnected in a square lattice fashion. The outer shape has a P-like footprint (Figure 1B, Figure S1, Table S1) to enable facile recognition by AFM. The inner pore lumen measures 7.5 × 7.5 nm^2^, and the ssDNA receptor is located within the lumen at 0, 3, or 6 nm depth when measured from the top pore rim (Figure 1A,B; Table S2). As another design feature, 22 biotin tags (Table S3) are located at the bottom of the DNA nanopore (Figure 1A,B) to achieve oriented binding onto a streptavidin-coated mica surface and make the channel lumen accessible from the top. The CanDo^69,70^ simulations suggest that the nanopore has a high degree of structural stability (Figure S2).

The DNA nanopore was self-assembled in DNA origami fashion by annealing a M13mp18 scaffold strand of over 7000 nucleotide (nt) length and a 5-molar excess of 158 specifically designed staple strands of 20–50 nt length (Tables S1, S2, and S3). Successful assembly of the nanopore was supported by a concise single band in agarose gel electrophoresis (Figure S3, lane 3) which migrated given its more compact size slower than the scaffold band (Figure S3, lane 2). The assembled pore was purified from quicker migrating staple strands (Figure S3, lane 3) by gel extraction (Figure S3, lane 6).

The correct shape and dimensions of the DNA nanopore were assessed via transmission electron microscopy (TEM) and AFM (Figure 1C and D, Figures S4–S6). TEM confirmed the P-shape of the pore (Figure 1C, left and right panels). The pore lumen measured 7.1 ± 0.4 nm (*n* = 21) in side length (Figure 1C, middle panel, Figure S4, Table S5) which is within the nominal width of 7.5 nm. The dimensions for the DNA nanoshell pore of 31.7 ± 1.3 nm (*n* = 15) and 22.4 ± 0.9 nm (*n* = 16) are matching the design (32.5 and 22.5 nm) (Table S5). By comparison, AFM revealed an outer pore width and length of 28.8 ± 1.9 and 39.8 ± 1.9 nm (*n* = 10), respectively (Figure S5, Table S5), which is larger than the expected dimensions of 22.5 nm × 32.5 nm. The larger AFM-derived dimensions are explained by sample spreading caused by AFM-tip compression, which is often observed for DNA nanostructures.^71,72^ In line with tip compression, the measured pore height was at 13.8 ± 2.4 nm lower than the nominal height of 20 nm. Due to the finite tip radius of the AFM tip of about 5 nm, the pore entrance was not structurally resolved.^73,74^

The presence of biotin tags (Table S2) at the pore base and their binding to streptavidin was confirmed via agarose gel electrophoresis (Figures S7 and S8). Streptavidin was titrated relative to a constant concentration of DNA nanopore. Streptavidin binding to biotin led to an upshifted gel band, attributed to slower migration of the bigger protein–nanopore complex, when compared to the unbound pore (Figure S7). Binding was also established by incubation with Alexa647-labeled streptavidin and tracking the increasing fluorescence intensity of the protein–DNA pore complex in the corresponding gel band (Figure S8). The molecular interaction mediated by biotin was furthermore confirmed by the oriented binding of the DNA nanopores on streptavidin-coated mica. DNA nanoshell pores were seen as clear islands (Figure 2C, Figure S6) protruding from the uniform streptavidin crystal layer.

**Figure 2:**
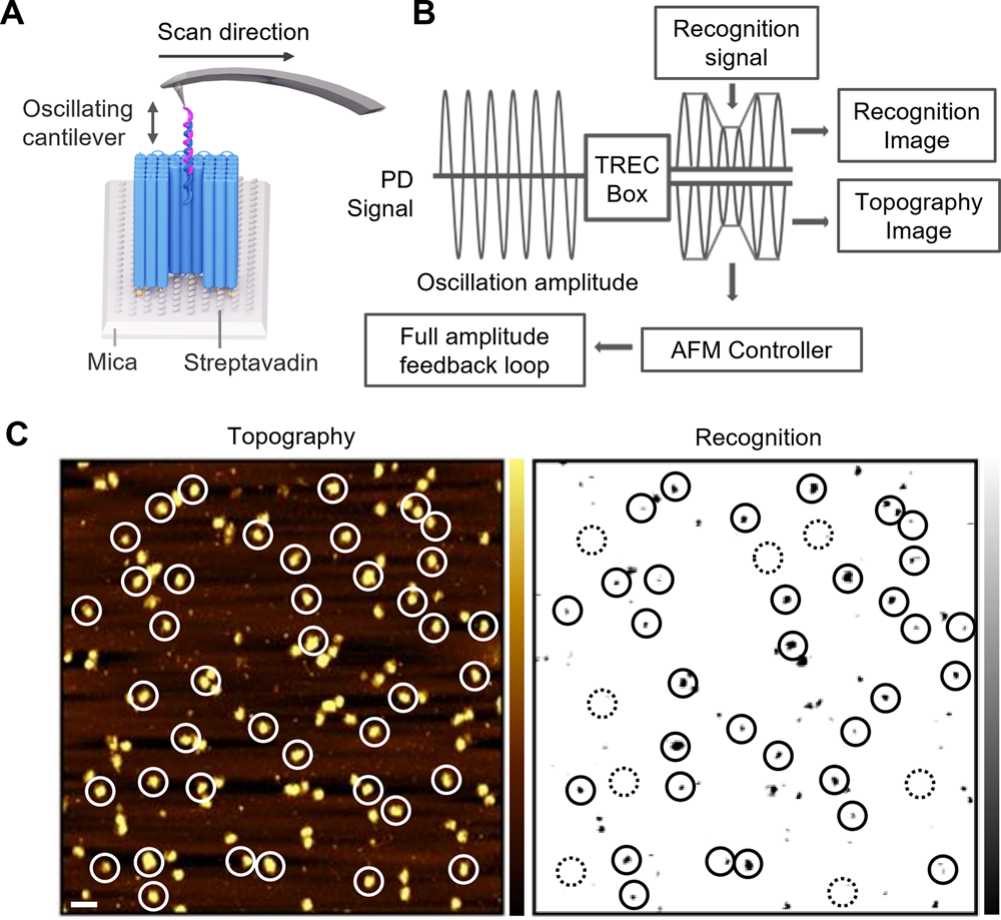
Molecular recognition of probe–receptor interactions within the DNA nanopore lumen. (A) Scheme on simultaneous topography and recognition (TREC) imaging measurements on the interaction between the probe strand tethered to the AFM tip and the receptor DNA strand tethered within the nanopore lumen. (B) Principle of TREC-based analysis of AFM oscillation data where PD is the photodiode signal from reading out the AFM cantilever deflection. (C) TREC images for topography (left) and recognition (right). Topography imaging revealed elevated DNA nanostructures (circled), while recognition images localized probe–receptor DNA interaction (circled with solid line). Topographical structures without specific binding interactions are indicated by dashed-line circles. The images were obtained using streptavidin-coated mica. Horizontal scale bar, 200 nm. Vertical scale of topography and recognition, 10 nm, and 0.1 V, respectively.

AFM-based topography and recognition imaging was used to investigate the selective interaction of the receptor with the cognate DNA probe inside the nanopore lumen (Figure 2A). For our analysis, the ssDNA probe was tethered via a flexible PEG linker to an AFM tip using established chemistry.^59,60,64^ Both receptor and probe strands are 20 nt long and complementary in sequence. In TREC, the specific molecular interaction of the probe and receptor strands was simultaneously mapped to the topography of the DNA nanoshell pore with nanometer precision. This was achieved by the scanning of the oscillating AFM tip over the sample surface (Figure 2A, Figure S9). The resulting amplitude oscillation from the tip–surface interactions was split into lower and upper parts to generate topography and recognition signals, respectively (Figure 2B). In the topography signal, upward bending of the tip, which is induced by the DNA nanoshell pore, reduces the amplitudes of the lower oscillation half (Figure 2B). By comparison, the recognition signal plots the reduced upward movement of the tip caused by the formation of a DNA duplex between the receptor and the probe strand. This reduced movement leads to lower amplitudes in the upper oscillation half (Figure 2B). The corresponding topography image shows elevations representing DNA nanopores (Figure 2C, left), while the recognition image represents probe–receptor binding as black spots (Figure 2C, right). Overlapping topography and recognition images display the binding efficiency to the lumen receptors (Figure 2C, spots circles with solid line).

The specificity of the interaction between probe and receptor strands was confirmed with several control experiments involving blocker and deblocker strands (Figure 3, Figures S10–S12). The blocker strand is complementary to the probe DNA and forms a DNA duplex (Figure 3A) as shown by gel electrophoresis (Figure S10, Table S4). When TREC analysis was conducted after addition of the blocker strand, the black-spot recognition signals for probe–receptor interaction disappeared (Figure 3C, middle) compared to before block (Figure 3C, left). This suggests successful blocking of the specific probe–receptor interaction. The corresponding topography signals were unchanged (Figure 3B), as expected. By comparison, the deblocker strand can remove the blocker via toe-mediated unzipping from the probe (Figure 3A, Figure S10, Table S4). Indeed, the TREC recognition image after deblocker addition showed the re-emergence of the black spots suggesting reinstated probe–receptor interactions (Figure 3B and C, right). These control experiments were conducted with the 0 nm DNA nanoshell nanopore. The hybridization took place in the presence of Mg^2+^ containing buffer and other monovalent cations which influence the interaction.^75−77^

**Figure 3:**
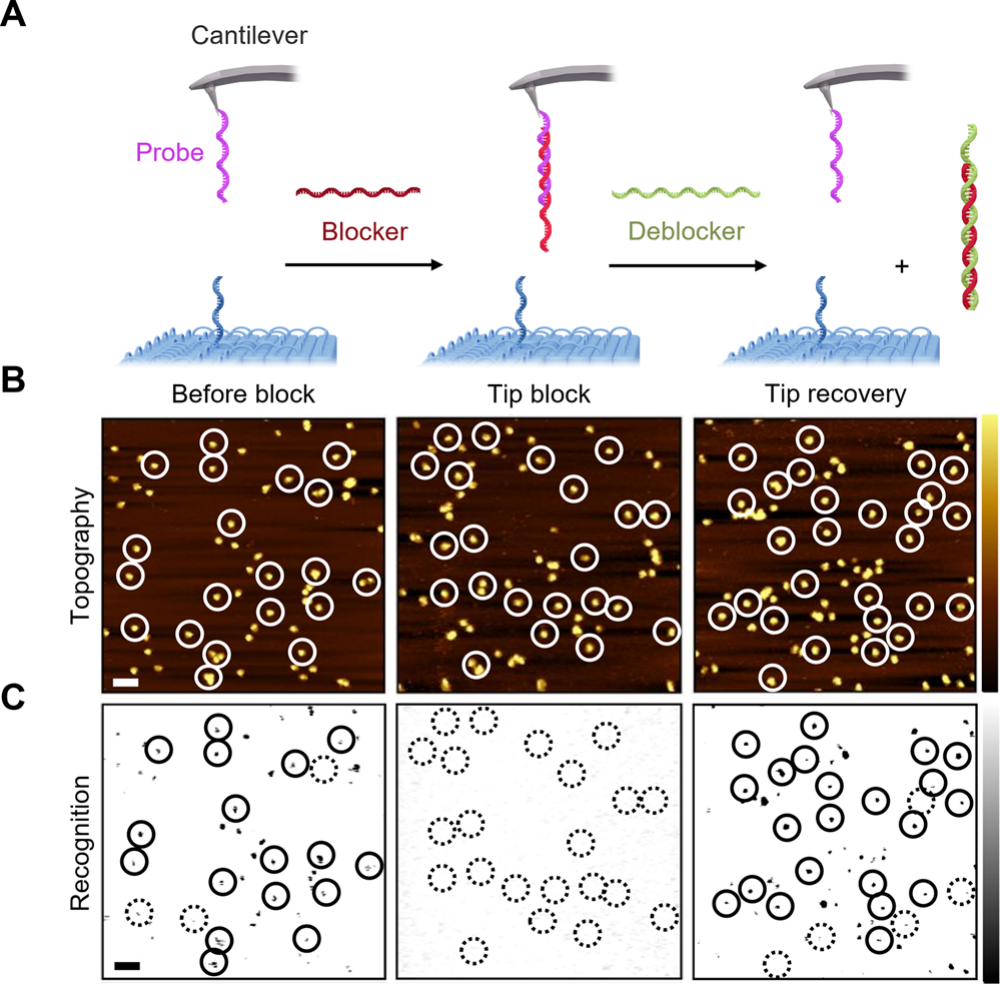
Probe–receptor interactions are specific. (A) Scheme on the blocking of the probe at the AFM tip by a blocker DNA strand (red), followed by unzipping the blocker strand with a deblocker DNA strand (green) of complementary sequence. (B) TREC analysis of the binding specificity of probe–receptor interaction. Topography images were recorded before tip block (left), during tip block (middle), and after tip block (right). (C) Simultaneously acquired recognition images were obtained before tip block (left), during tip block (middle), and after tip block (right), showing recognition before tip block (left) and after (right) but not upon addition of the blocker strand (middle). Circles with solid and dashed line are defined as in Figure 2. The images were obtained using streptavidin-coated mica. Horizontal scale, 200 nm. Vertical scale of topography and recognition, 10 nm and 0.1 V, respectively.

After establishing specificity, we examined how the molecular interaction was influenced by the vertical position of the receptor strand within the channel lumen. We compared the interaction for nanopores with receptors at 0, 3, and 6 nm vertical depth from the pore top (Figure 4A). In TREC read-out, the recognition images revealed molecular interaction for all constructs (Figure 4B); the topography images show the expected elevations caused by DNA nanopores (Figure S13). In detailed analysis of the recognition spots’ size, its mean radius, Rs, was found to depend on the receptor depth. With increasing receptor depth (0, 3, and 6 nm), the Rs values decreased from 22.7 ± 2.3 nm over 21.2 ± 3.4 nm to 16.7 ± 3.2 nm, respectively (Figure 4B). This dependence of the recognition spot size is expected when considering the molecular nature of the interactions between the AFM probe and the nanopore receptor. In particular, the deeper the position of the receptor, the longer the linker DNA polymer strand needs to thread inside the lumen to reach the receptor (Figure 1A). This leads to shortening of the probe/receptor/linker part outside the pore lumen, and this length correlates with the recognition spot size.^64^

**Figure 4:**
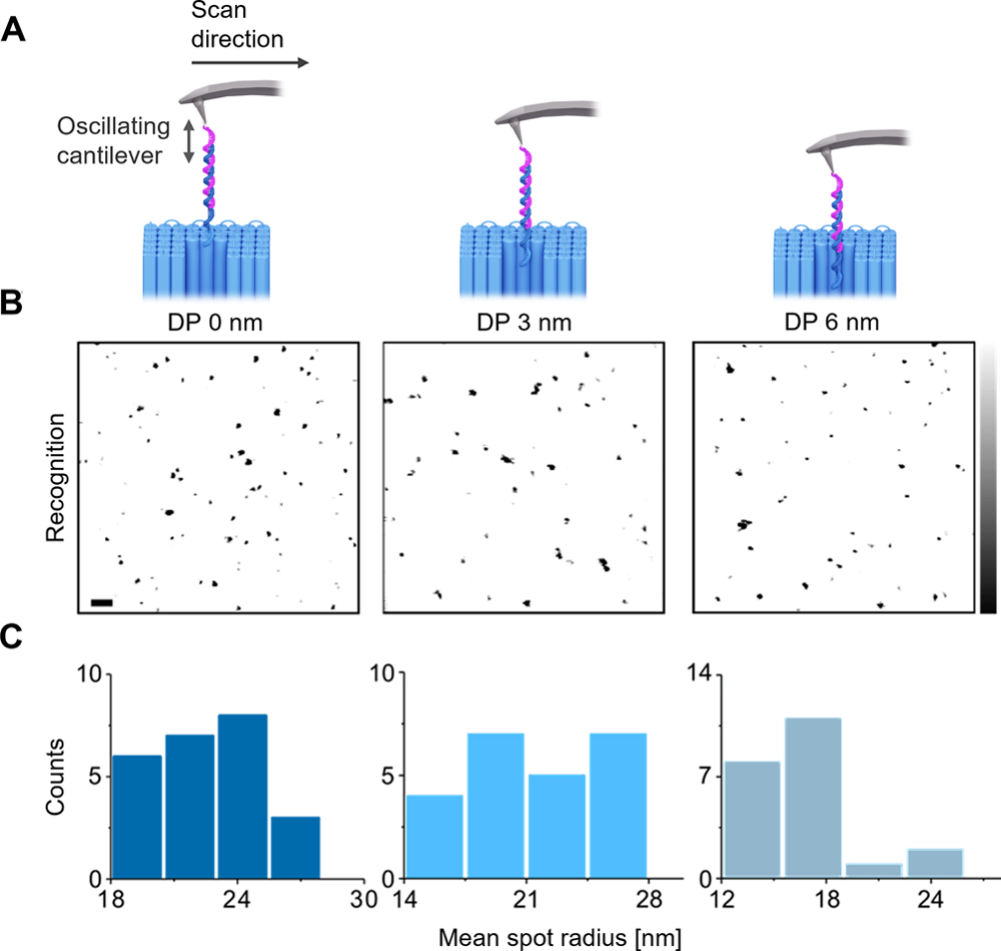
Probe unbinding depends on the receptor’s depth position as analyzed with recognition imaging. (A) Schematic illustration of probe–receptor binding at receptor positions at the pore entrance (DP-0 nm), 3 nm depth (DP-3 nm), and 6 nm depth into the lumen (DP-6 nm). (B) Recognition images for DNA nanopores at the different binding sites. Horizontal scale, 200 nm. Vertical scale, 0.1 V. (C) Plots summarizing the statistical distribution of the mean radius of recognition spots for the three recognition sites. Small aggregates resulting from multiple pores were not considered for this analysis.

Single-molecule force spectroscopy (SMFS) gave insight into the strength of binding as well as the dissociation and association kinetics. In SMFS, the probe-tethered cantilever tip is approaching the receptor-modified pore, physically contacts the sample, and is then retracted (Figure 5A, 5B). The interaction force, which is proportional to AFM cantilever deflection, was simultaneously monitored as a function of the distance of the AFM tip and nanopore, as determined by piezo movement. When the approaching tip contacts the pore (at 0 nm), the force increases (Figure 5B, light blue trace), which reflects the mechanical resistance experienced by the tip (Figure 5B, light blue trace). By comparison, in the retraction, successful molecular interaction between probe tip and receptor pore first leads to a downward bending of the tip (Figure 5B, dark blue trace). Ultimately, the probe and receptor interaction ruptures and the force signal switches to the zero-position of the normal trace (Figure 5B). The strength at which rupturing occurs (Figure 5B, downward spike) is the dissociation force.

**Figure 5:**
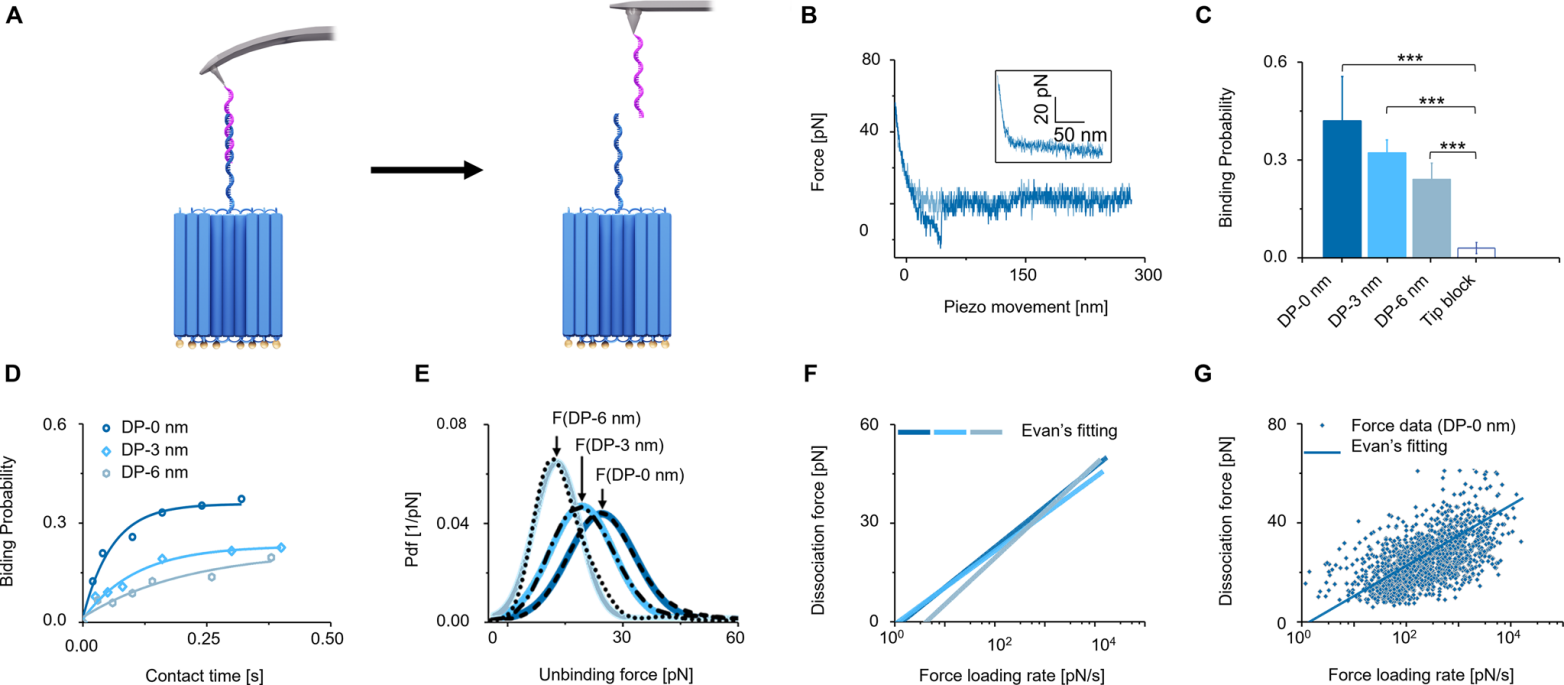
Single-molecule force spectroscopy and determination of binding kinetics of the probe–receptor interaction within the DNA nanopore confinement. (A) Scheme on the binding of the cantilever-tethered probe to receptor DNA and its rupturing by AFM tip retraction. (B) Sample force–distance curve showing the interaction between probe–receptor DNA leading to an unbinding event. Light blue corresponds to the approaching curve; dark blue curve shows the retraction curve. Rupture signals were only seen in the retraction curves. The inset shows force–distance curves resulting from the tip block without rupture events. (C) Binding probability at different receptor sites at a scanning velocity of 75 nm s^–1^. (D) Plot of the binding probability in dependence of the contact time for the three receptor sites, with mono-exponential fitting. (E) Experimental probability density functions (PDFs) for all three receptor sites. The dashed black lines correspond to the force data of individual pore constructs. The different shades of blue indicate the Gaussian fits of the PDFs (equivalents of continuous histograms). The peak of the fits corresponds to the most probable unbinding force, *F*, with its standard deviation measured at a scanning velocity of 300 nm s^–1^ (Supporting Information). (F) Plot on the dependence of unbinding force versus force loading rate for probes dissociating from the receptor DNA strand. The line plots show the Bell–Evans single-bond fit for probe–receptor interaction at the binding sites of 0, 3, and 6 nm. The corresponding data points including their fits are presented in Figures 5G, S14A, and S14B for the receptor sites placed at 0, 3, and 6 nm, respectively. (G) Scatter plot of the loading rate against the unbinding force for probe–receptor binding at the pore entrance (DP-0 nm).

Our SMFS data show that the probability of successful probe–receptor binding per force–distance cycle, termed binding probability, decreased with increasing receptor depth (Figure 5C). This is consistent with an elongated diffusion path of the probe to the receptor site in the lumen. In a control experiment, the binding probability strongly decreased in the presence of the blocking strand (Figure 5C), suggesting that the measured binding events represent the specific molecular probe–receptor interaction. We also monitored the binding probability at different approaching/retracting velocities. The results show that the binding probability increases for lower velocities and corresponding longer contact times between tip probe and receptor nanopore (Figure 5D). This is in line with kinetic considerations. The binding probability was found to be higher for the 0 nm receptor position and dropped for lower receptor positions (Figure 5D) which reflects the reduced accessibility of the receptor at lower channel positions. Binding probabilities at all binding sites were typically below 50% under the experimental conditions.^62,78^ Nevertheless, recognition and binding to the tethered receptor strand were detected in almost all pores, as shown by the previous TREC measurements.

The SMFS data were analyzed to obtain the kinetic rate constant for duplex association, *k*_on_. The kinetic plots (Figure 5D) were first least-squares fitted for a monoexponential rise to extract the characteristic contact times, τ, for the binding interaction (Table 1). The kinetic rate constant *k*_on_ was then calculated from τ and the effective probe concentration assuming first-order binding kinetics. The resulting *k*_on_ values (Table 1) reveal significant differences (*p* < 0.05) between the receptor sites at the entrance (0 nm) and at 6 nm. Comparing the association kinetics and binding probabilities for the different receptor positions showed a reduction in *k*_on_ and binding probability for deeper receptor sides, which is plausible considering the steric constraints for duplex formation in the nanoconfinement of the channel lumen. The surface coverage of the DNA nanoshell pores was assumed to be constant for all DNA nanopores. Our kinetic on-rates are in line with previously published data for comparable DNA hybridization interactions estimated with different techniques.^79−85^

**Table 1:** Kinetic Property of Probe DNA Binding to Probe Receptor DNA within the Shell Nanopore^a^

Construct	τ [s]	*k*_on_ [× 10^4^ M^–1^ s^–1^]	*k*_off_ [× 10^–1^ s^–1^]	*x*_β_ [Å]
DP-0 nm	6.1 ± 2.7	8.7 ± 2.2	1.6 ± 0.7	9.2 ± 1.5
DP-3 nm	5.1 ± 2.6	5.4 ± 2.3	2.0 ± 1.0	9.4 ± 2.3
DP-6 nm	2.0 ± 2.1	3.1 ± 2.0	5.1 ± 5.4	9.1 ± 2.3

a From single-molecule force spectroscopy, the kinetic off-rate and on-rate *k*_off_ and *k*_on_, bond lifetime τ, and width of the energy barrier *x*_β_ were determined.

SFMS data also yielded the dissociation forces of the molecular interaction in dependence of the receptor position. The average forces were ∼26 ± 6, 22 ± 4, and 19 ± 8 pN for receptor sites at 0, 3, and 6 nm, respectively (Figure 5E). Our force values are in close agreement with previously reported AFM data on duplex interactions of isolated 20 basepair (bp) DNA strands.^62,86−89^

To fully explore the energy landscape of the force-driven dissociation path, we obtained the kinetic rate constant for dissociation *k*_off_ and *x*_β_, the width of the energy barrier for dissociation. The parameters were determined by varying the retracting velocities in the force–distance cycles followed by computing the dissociation force vs loading rate (retracting velocity time effective spring constant) relation with the maximum likelihood approach (Figure 5F,G). The scatter plots of individual dissociation forces as a function of their force loading rate were fitted with the Bell–Evans single energy barrier model (Figures 5F, 5G, and S14) to derive the *k*_off_ and *x*_β_ values (Table 1).

The *k*_off_ values are in line with previously reported data for a 10 bp duplex determined using AFM-based SMFS and dynamic force spectroscopy.^90^ Our *k*_off_ values differ by 1–2 orders in magnitude from several reported on DNA hybridization using a quartz crystal microbalance and surface plasmon resonance.^80,91^ The difference can be attributed to surface adhesion effects connected with these techniques or DNA rebinding which is disregarded in our single-molecule force spectroscopy.^92^

The *k*_off_ value was significantly higher for the 6 nm than for the other two receptor positions (Table 1). In general, differences in *k*_off_ reflect a change in dissociation barrier upon transition from bound to unbound state along the direction of the applied mechanical load. In the case of receptor–probe interaction, this might arise from an incomplete binding to receptor sites at deeper positions in the lumen. For example, a loss of one base pair in the formation of duplexes for DNA strands of comparable length leads to a dissociation force decrease of about 4 pN, as shown in a previous study.^93^ Our observed force differences might also be caused by slightly different directions of the pulling force during AFM tip retraction. Dissociation forces are known to depend on the direction of the pulling force, and rupturing the tethered duplex can occur either by unzipping or in shear mode.^94,95^ Due to the high flexibility of the DNA duplex, pulling by the AFM tip is likely accompanying unzipping by a distortion of the duplex structure. However, unzipping from receptors at lower channel depth is expected to lead to more overall structural distortion resulting in more peeling-like than zipper-like dissociation.^94−97^

## Conclusions

In this study, we have explored molecular recognition in confinement by pioneering the synergistic combination of DNA nanotechnology and single-molecule force microscopy. DNA nanotechnology offers exquisite molecular control over nanoconfinement dimensions and receptor properties, while AFM reveals detailed insight into interaction forces. In addition, information about binding and dissociation kinetics can be retrieved from varying the parameters in force spectroscopy experiments. We chose this unique path to improve the basic understanding of molecular recognition in nanoconfinement and provide a basis for rationally engineering nanopore sensors and filtration devices. Previous work on nanoconfinement explored DNA hybridization in protein pores with single-channel read-out.^16,22^ Detecting molecular interaction in nanopore confinement^22,98,99^ is highly tunable and attains specificity by including ligands that target molecules of interest.^23,24,100,101^ Nevertheless, in the protein pores, the receptor position and lumen dimensions were fixed, the interaction of the DNA probe of 6 nt length to form a duplex could only take place in one orientation as the pore lumen was only 3 nm tight and high, and force-induced unbinding could not be studied.

In our study, we took advantage of the design flexibility offered by DNA nanotechnology^30−39^ and generated a nanopore with a cross-sectional lumen area greater than 50 nm^2^. The lumen was long and wide enough to accommodate DNA receptor strands of 20 nt length at three distinct *z*-positions and multiple orientations within the lumen. This greater design scope was essential to reveal how molecular interaction between the DNA probe and receptors depends on confinement. While the probe–receptor interaction was expectedly specific, our single-molecule analysis revealed that strand dissociation forces and the kinetic off-rate were lower when duplex rupturing took place deeper inside the channel lumen. These data strikingly underscore the effect of nanoconfinement on molecular interactions. Furthermore, we also quantified how the association rate for duplex formation is hindered by confinement. With the receptor anchored deeper into the pore lumen, it can be assumed that part of it is located inside and the other part outside of the pore. Therefore, at deeper sites, the binding kinetics rates are likely modulated by several factors including restricted steric movement, binding orientation, and nanoconfinement as opposed to freely mobile DNA.

Due to the flexible anchoring of the AFM probe DNA via the PEG linker, we expect that unbinding of cargo-receptor DNA at the pore entrance (0 nm) occurs through unzipping. However, at deeper recognition sites within the lumen, contributions from shearing will likely appear, because of the constrained mobility of the cargo-receptor duplex within the lumen, as well as the complexity of dehybridization due to restrained orientation. Our findings are of direct relevance in fields where molecular recognition takes place in nanopores, such as nanopore sensing, synthetic biology to form a biomimetic version, and bioaffinity filtration with porous membranes. More insight into nanoscale effects may be gained in the future by studying different probe analytes such as globular proteins, binding to multiple receptors, thereby opening up the exploration of molecular transport, but also by using other, high-throughput read-out methods based on fluorescence^41^ and modeling approaches.^102^ In conclusion, our study has contributed to a better understanding of molecular recognition in confinement and has laid out tools that facilitate more discoveries for basic and applied science.

## Supporting Information Available

Experimental section and additional supporting material including figures on the design, connectivity, thermal fluctuation analysis, PAGE characterization, gel electrophoretic mobility shift analysis, TEM and AFM imaging, and schemes (PDF)
