# Supplementary material for: Molecular Recognition in Confined Space Elucidated with DNA Nanopores and Single-Molecule Force Microscopy
Source: Nano Lett. 2023 May 11;23(10):4439–47. doi: 10.1021/acs.nanolett.3c00743 (PMC10214486; doi:10.1021/acs.nanolett.3c00743)
Supplement: Supplementary file 1 — nl3c00743_si_001.pdf [file nl3c00743_si_001.pdf]

Supporting Information

Molecular recognition in confined space elucidated with DNA nanopores and single-molecule force microscopy

Saanfor Hubert Suh*, Yongzheng Xing**, Alexia Rottensteiner**, Rong Zhu*, Yoo Jin Oh*,

Stefan Howorka**, Peter Hinterdorfer*

* Department of Applied experimental Biophysics, Institute of Biophysics, Johannes Kepler University Linz, Gruberstraße 40, 4020, Linz, Austria

** Department of Chemistry, Institute for Structural and Molecular Biology, University College, London, London, WC1H0AJ, England, UK

Table of Contents

1. Experimental section
    1.1. Materials
    1.2. Design and assembly of the DNA nanoshell pore
    1.3. Agarose gel electrophoresis (AGE)
    1.4. Agarose gel purification
    1.5. Atomic force microscopy (AFM)
    1.6. Transmission electron microscopy (TEM)
    1.7. Polyacrylamide gel electrophoresis (PAGE)
    1.8. Probe hybridization to AFM tip
    1.9. Topography and recognition (TREC) imaging
    1.10. Single molecule force spectroscopy
2. Supplementary tables and figures
3. Note A, TREC analysis of probe-receptor DNA binding
4. Note B, PDF analysis of unbinding forces
5. References

1. Experimental section

1.1 Materials

All DNA oligonucleotides were purchased from Integrated DNA Technologies (IDT DNA, Belgium), and dissolved in highly pure nuclease-free Hyclone water from GE healthcare (Utah, USA). The viral M13mp18 DNA scaffold was purchased from Tilibit nanosystems (Frankfurt, Germany). Ultrapure Tris, $NiCl_2$, $MgCl_2$, TAE and TBE buffers were acquired from Sigma-Aldrich (Sigma Aldrich, Germany). Freeze "N" Squeeze DNA gel-extraction spin columns and precast PAGE gels were bought from Bio-Rad Laboratories Inc. (USA). Muscovite mica (Nano-Tec V-1 grade) was purchased from "Christine Gröpl Electonenmikroskopie" (Tulln, Austria). Ultrapure millipore (MilliQ grade, Millipore, Austria) water with resistivity > 18.2 MΩ x cm was used for all buffer solutions and for rinsing sample surfaces and other apparatus used during the measurements. AFM cantilevers were obtained from Bruker (Bruker, USA). Buffers for tip chemistry were prepared using 20 x SSC (Thermofischer, Germany), SDS, glycerol (Sigma Aldrich), $NaHCO_3$ and ultrapure Tris buffer (Sigma Aldrich). AFM cantilevers (MSCT) were obtained from Bruker (Bruker, USA). When required, the pH was adjusted by the addition of 1M NaOH or 36 % HCl.

1.2 Design and assembly of the DNA nanoshell pore

The nanoshell pore was designed in caDNAno in a square lattice configuration (Figure S1). The M13mp18 scaffold DNA strand was used to assemble the DNA nanoshell pore together with staple strands (Table S1 and S2). 22 biotin tags for streptavidin interactions were added at the base of the nanoshell pore (Table S3). To fold the nanoshell pore, M13mp18 was mixed in 1:5 ratio with the staple strands in 1x TAE buffer (20 mM tris (hydroxymethyl)aminomethane (Tris) base, 10 mM acetic acid, 0.5 mM ethylenediaminetetraacetic acid (EDTA), pH 8.3) supplemented with 14 mM $MgCl_2$. The DNA nanostructure was assembled using a 42 h folding protocol, including heating at 75 °C for 10 min to denature undesired DNA secondary structures, and then slowly annealing from 65 °C to 25 °C using the thermal ramp from 65 °C to 25 °C using a temperature drop of 1 °C/, followed by cooling from 25 °C to 10 °C at at a rate of 1 °C per 5 min, and storage at 4 °C.

1.3 Agarose gel electrophoresis (AGE)

The assembled DNA nanoshell pores (5 nM) were analysed with 1 % agarose gel electrophoresis in 0.5 X TAE buffer (supplemented with 10 mM $MgCl_2$, pH 8.0). The gel was run at 65 V for 90 min in an ice-bath. The bands were visualized by ultraviolet illumination after staining with GelRed solution. A 1kb marker (NEB) was used as the reference standard molecular ladder. After electrophoresis, the gels were imaged with a gel imaging system (ChemiDoc MP imaging system, BioRad) and analyzed with the imaging lab software.

1.4 Agarose gel purification

After folding and electrophoresis, all bands of DNA nanoshell pores were cut out from the gel. The desired bands were physically excised, chopped into smaller pieces, transferred into 1.5 ml Eppendorf

tubes, and stored at -20 °C overnight to increase purification yield. The gel slices were further crushed into smaller pieces and filtered through "Freeze 'N Squeeze DNA extraction" spin columns at 13,000 x G for 3 min at room temperature.

1.5 Atomic force microscopy (AFM)

For AFM analysis of the nanostructures, the DNA nanoshell pores were adsorbed onto mica following a published procedure which uses Ni^{2+} cations for immobilization by bridging the negative charges of DNA and the mica substrate[6,7]. Briefly, onto freshly cleaved mica, a solution of DNA nanoshell pores (~ 5 nM in 1x TAE, 10 mM $MgCl^2$, 25 µl) with imaging buffer (1x TAE, 14 mM $MgCl_2$, 4 mM $NiCl_2$ pH 7.4, 75 µl) was added and incubated for 5 min. The mica surface was then rinsed with the imaging buffer (total volume of 1ml) and imaged with AFM in tapping mode. The AFM topography images of the pores were acquired in fluid at room temperature using a PicoPlus 5500 AFM instrument (Agilent Technologies, Chandler, AZ) equipped with a PicoScan controller (Molecular Imaging, Tempe, AZ) operated in tapping mode using non-functionalised MSNL-10 cantilevers (Bruker, USA) with nominal spring constants between 0.1 – 0.6 N/m. Cantilever oscillation frequencies were set between 10 - 30 kHz, with the free amplitude usually less than 5 nm to minimize imaging force on the nanostructures. Imaging was performed at scan rates between 1 – 1.7 Hz with 512 pixels per line scan.

For geometrical analysis of selected DNA nanoshell pores, the image scan sizes were kept to 3 µm x 3 µm or less at a resolution of 512 pixels. Images were processed using Gwyddion 2.5 (http://gwyddion.net/) for line-by-line flattening and removal of tilt using first, second or third order polynomials. Selected individual nanoshell were cropped from the original image for illustration.

1.6 Transmission electron microscopy (TEM)

The DNA origami structures (6 µL) were added onto glow discharge-treated TEM grids (Agar Scientific, AGG2050C) and stained with a 2 % uranyl formate solution. The samples were analyzed on a JEM-2100 electron microscope operated at 200 kV, and images were acquired with an Orius SC200 camera.

1.7 Polyacrylamide gel electrophoresis (PAGE)

To verify the specificity of the probe-receptor DNA interaction implemented via the toehold-mediated strand displacement mechanism, 10 % precast native PAGE gels in 0.5 x TBE running buffer supplemented with 10 mM $MgCl_2$ solution were used. DNA strands (5 µl per sample) in 1 x SSC buffer containing 14 mM $MgCl_2$ were mixed with 5 µl 6 x gel loading dye before transferring the individual solutions into the wells. A 100 base pair marker was used as the reference molecular ladder, and the gel was run at 70 V for 1 h in an ice-cold bath. After the electrophoresis run, the gels were post-stained with GelRed for 10 min and visualized using a Chemidoc MP imaging system.

1.8 Probe attachment to AFM tips

MSCT AFM tips were functionalized with probe DNA as described using an established protocol[3]. Briefly, in first step of the modification protocol, the AFM tips were amine-functionalized with APTES using a

vapor phase deposition method. Subsequently, the heterobifunctional crosslinker NHS–PEG_{24}–aldehyde (creative PEG works, USA) was covalently bound to the amine groups on the tips for 1 h. For the conjugation with probe DNA, the AFM tips were immersed in 1x SSC buffer (15 mM sodium citrate, 150 mM sodium chloride) containing 250 µM 5'-/5AmMC6 – linked probe DNA (25 µl) and 0.5 µL of sodium-cyanoborohydride, 1 M $NaCNBH_3$ solution (13 mg $NaCNBH_3$, 20 µL of 100 mM sodium hydroxide in 180 µL millipore water) for 3 – 6 h. Subsequently, 10 µL of 50 mM Tris – HCl was added to the solution to quench the remaining aldehyde groups. After 10 min reaction time, the tips were washed with $NaHCO_3$ buffer (three times for 5 min), 2x SSC supplemented with 0.1 % SDS (three times for 5 min), 0.2x SSC supplemented with 0.1% SDS (three times for 5 min), and millipore water (three times for 5 min) to be stored dry in sealed Petri dishes at 4 °C.

1.9 Simultaneous topography and recognition (TREC) imaging

AFM TREC imaging in fluid was performed by acoustic excitation using an Agilent 5500 AFM (Agilent, Chandler, AZ, USA) with MSCT cantilevers with nominal spring constants between 0.1 - 0.6 pN/nm. The topography and recognition data were recorded using a commercially available electronic unit (Picoplus with PicoTREC from Molecular Imaging, Tempe, AZ, USA).

The AFM instrument was set up before use to calibrate a laser beam reflected of cantilever back onto a position-sensitive photo diode. The samples were imaged in a liquid cell with tapping mode using MSCT cantilevers E and F of with higher spring constants to provide better resolution. During imaging, the cantilever scanned using the faster x and the slower y scanning direction. 512 lines per image were used for all AFM measurements. The cantilevers were oscillated at sweep at low frequencies between 2 - 50 kHz with lower frequencies used to minimize cross-talk due to the extra time required for the topography signal[4]. Imaging was performed at scanning frequencies of between 1 - 1.7 Hz lines per s. The force amplitude setpoint was adjusted during imaging. Integral (I) and proportional (P) gains were initially set to 1.0 and varied during imaging. All TREC measurements were performed in scanning areas from 3 µm x 3 µm to 0.5 x µm 0.5 µm using full amplitude feedback[4]. The AFM instrumentation was checked by performing imaging on bare mica in imaging buffer. All measurements were operated in liquid environments in the AFM liquid cell using an AFM small scanner. All measurements were performed in hybridization buffer containing 50 mM Tris, 50 mM NaCl, 50 mM KCl, 5 mM $MgCl_2$, pH 7.9.

1.10 Single molecule force spectroscopy

Single molecule force spectroscopy measurements were performed with a Picoplus 5500 AFM setup equipped with an AFM controller using MSCT AFM tips with nominal spring constants between 10 – 30 pN/nm. All AFM measurements were performed in hybridization buffer containing 5 mM Mg^{2+}. At the beginning of measurements, freshly prepared sample surfaces containing DNA pores attached to streptavidin were checked using AFM in tapping mode to ensure high surface coverage. AFM imaging of the surface was performed with MSNL cantilevers E or F in the hybridization buffer at room temperature.

Force spectroscopy experiments were conducted in contact mode using MSCT cantilevers functionalized with probe DNA. Force–distance (FD) cycles were acquired for DNA nanoshell nanopores

at room temperature in hybridization buffer containing 50 mM Tris, 50 mM NaCl, 50 mM KCl, 5 mM $MgCl_2$, pH 7.9. At a fixed lateral position, the modified cantilever was approached (trace) to the surface and subsequently retracted (retrace) at varying velocities corresponding to 0.5 s/cycle, 1 s/cycle, 2 s/cycle, 4 s/cycle, 6 s/cycle and 8 s/cycle (scanning velocities of 1250 nms^{-1}, 577 nms^{-1}, 300 nms^{-1}, 150 nms^{-1}, 100 nms^{-1}, 75 nms^{-1} for sweep range of 300 nm respectively). 1250 nms-1, 577 nms-1 correspond to the fast scanning regime, 300 nms^{-1}, 150 nms^{-1} correspond to the mid-range scanning regime and 100 nms^{-1}, 75 nms^{-1} correspond to the slow scanning regime. The deflection of the cantilever in volts was monitored by the laser beam incident on the backside of the cantilever and was plotted against the tip-sample separation distance in nm (z-position of the piezo scanner) and from this relationship, the force was calculated. FD cycles were obtained at low indentation forces (0.04 – 0.1 V maximum force limit) corresponding to ~ 50 – 100 pN to minimize adhesive or unspecific events. All force spectroscopy experiments were performed by varying the force loading rate defined as the product of the pulling velocity (v) and the effective spring constant (k_{eff}) by varying v from 75 nm/s to 1250 nm/s. In light of the small size of nanoshell pores, small sweep ranges of 300 nm with sweep rates between 0.5 – 8 Hz (0.5 s/cycle – 8 s/cycle) were applied resulting in loading rates from ~ 10 to 5000 pN/s. Typically, 200 to 600 force-distance cycles were recorded per DNA construct and per cantilever at a set loading rate. For ease of data interpretation and determination of kinetic association rates, single functionalized tips were used for the three constructs. After each set of force measurements, the sensitivity and the spring constants were determined. For sensitivity measurements, freshly cleaved mica was mounted on a sample plate with the liquid cell filled with 600 µl of hybridization buffer at room temperature. For each sensitivity measurement, 100 FD curves were obtained using the functionalized tip at sweep rates of 1 Hz (1 s/cycle) on random areas of the freshly cleaved hard mica surface. These curves were used for data analysis of probe DNA binding. The calibrated spring constants were obtained using the Keysight Thermal noise measurement program in the PicoView software. Furthermore, the specificity of the binding studies was also determined by performing tip block experiments via the TMSD mechanism and verified during TREC experiments.

2. Supplementary tables and figures

Table S1. Sequences of DNA staple strands used for the assembly of the DNA nanoshell pore.

ID	Sequence 5′ → 3′
HShell001	AACTGGCAATTCATTAGAACCTTTAAATCACCAAC
HShell002	ACATTAAATGTGAGCAAACGGCTAGTAAAAATCG
HShell003	CAAAGGCTATCAGGTCGCCGGAGATGAGATGGGCCC
HShell004	ATACCCAAAAGCCTGTTTAGGGCTTTCACACGACC
HShell005	GAACAAGAGTAAGCTCATTCCTCATTTTCGTCACCAGTATACC
HShell006	CGCTTGCTTTCCCCCACTACGAAAAACC
HShell007	TTAGAGCTTAATTGCTGAGGTCAGGGCAC
HShell008	CCATTCGGTCATTAATCAGTTGCTATTTAACG
HShell009	GTTATCTTCGCTAAAGGGGGTAGTCAGATCAA
HShell010	CCTGCCGGAAAAATCAAGTTTGCCTTTAGCGTACCAGTAGGTTTTGA
HShell011	CATCAAGAAGCAAAAGCCAAATAAAACAGCCATTTA
HShell012	ACCCTTCTTTGATATAATCATTCTGGCCTTCTGACCTAATGCGC
HShell013	TCCTGTAAAACATGAATAATGGAAGGGTCAAT
HShell014	AATCAGCAAGAAATATAACTGACAAAGAACGC
HShell015	TCTTTATCCCTCATATATTTTATAT
HShell016	AATGAAAATTAATTACATGGAAACAGTACATATGTA
HShell017	CCTCAGGAAGATCGCACAGCTAACTCGGAAGCAAGAGGCGG
HShell018	TAATTATGACCAAAAACATTATGACCCTGTTTA
HShell019	CCTGACCGGTAATATCCAGATTTCCCAGTCACGACGACCGACCGATAC
HShell020	AACGTAGAAAATGCCCACGCGTAATAAGGAAAGCG
HShell021	TTTTAAGAAGGCAATCGCAAATATGTAA
HShell022	ATACAGTAACAGTACCCTGATTGCACAAAATAGAAAC
HShell023	AATGAGTGTCCAGCCATCTGGTGC
HShell024	TCATTTTCGAGGAAATCAGGTCTTTTACGCGA
HShell025	GTAAGAATATTAGACAGACCGAGTAGTATAACGTAAT
HShell026	TTAGAGCCGCCACTCAGAGCCGCCACCAAGGA
HShell027	ATAAGCGTTTTATCAGATATAGAGCCAGGTGAATTAATTG
HShell028	TTGTTTGCTTTGTCCAGTCGTTGCGTT
HShell029	TTTAGTATTAAACTTTCCTTTTAGGAGCCAAATCA
HShell030	ATTAGCAAGCCCGACTTGCGGGGAATATGATATTC
HShell031	GGTTAGAATTGAGTTAAAAATGAAAACATAAT
HShell032	GCAAGCCAAAAGACCAGCGCAGTAAATGAATT

HShell033	TCAATAAGGCTTTTTAATTTACTACAACAGTTTTGTGTAT
HShell034	TTCTCGTCTTTCCGAACTAAGAAGAAAATATAACA
HShell035	GAGCATATCATTTTGCTTATCAGACAAGCAAAGCGAA
HShell036	AGGTAAATTCACCGTCTCGGCTGTCCAA
HShell037	AATGAAACCTGGGAATTAGAAGGCTAAGCAAGCGAAA
HShell038	GCAAACTCCAACTAATGGTTTCGGAACGCGGT
HShell039	CGAGTCCTGATTATCATCATATTCCTGAGGAACAAACGTT
HShell040	CGTACACATCACGCAAATTAGCAACTGTGGTA
HShell041	GGCGTCTTCTGACCTAAATTGCAAATCCTACAGAGGCTAGCCC
HShell042	GGGAATTCAACTAGGCATAGCCTGTAGCCA
HShell043	AAGAGCTCAGTAACTAAAGAAGACAGCATGACCGA
HShell044	ACCATCAGAGCCACTCAGGAGGTTTAGTATAG
HShell045	GAGCACCCAGACTGTAGCGCAACGAGCG
HShell046	ACGCTATGCGTTTGTGATAAGATATATTAGGG
HShell047	CAACCTTTGAGGCGCCGCCATGAAACATGAAA
HShell048	CCACTATTAATTTTAAAAGTGACAACTCATAATAC
HShell049	ATTTGAGGAAAATATCTATCATTCCTCCCATCCAAAT
HShell050	TGAATTTATAAGACGCAGAGAGATAGAG
HShell051	ACAGTTGAATTGCTGAAAAATAATATTCA
HShell052	AGCTATATTGAGTAGATAAACAAAGGCAA
HShell053	CAGTCTCTGCATTAAAGAACAAGTAAGCAGATATAAT
HShell054	GATAGCCCCGCTACAGCGGGCGCTTATCGGCCT
HShell055	GCATTAACATCCTTCATTTGGGCGCAGAGCCT
HShell056	GTGTAATTGTGTCAAGCGCGTTAGTTTGTCCCAAT
HShell057	CGATAGTTTAACAGTGATGCCCC
HShell058	AGTTGCACCGTTGTACTC
HShell059	ACCAGGCGTTGCGGGAGCAATAA
HShell060	CCGGGTAGAGAGTTATTGCCCTAAATCGGCCAAAAGAATAGCCCGAAGTG
HShell061	ACGGTAATTTACAACAAGAACCTCAAATTGAGAGC
HShell062	AAACAGGGCGCTAAGAAAGCGCCCCCGATTTAGAGCTTGACGGG
HShell063	GAGCCACCACCGGAACCGCCTCACCGGACATTCAAA
HShell064	TAATTTTCCCTTTGAATTACGTATCATCCGGTCAATACCA
HShell065	GCGCTCACTCGTGGCACGCCATTCAGGCATTTTAGCC
HShell066	TATCGGTACTTGCCAGACGAGAGGCTCTTTCATCA
HShell067	ATGGATTGCGAATAATACAAAATAGGGGGGTAAGGAT

HShell068	ATTTGAATTACGAATGCAGAAGGTTAAATCAGCTC
HShell069	GAGTATTAAGAGGAAGCAATTTGTAAAACAAG
HShell070	CATTGAGCCCAGTGGGATAGGTCACGTTGGTGTTTTG
HShell071	CGGTTTTCATCCATTAAAGCAAAATCACCAGTAGC
HShell072	TCGGAGGCAAAGATACGAGCCACATTAAGGAAACC
HShell073	AATCGTCTGGTAGAAGAAGCAATACGCCG
HShell074	AAATAGACTTTACAAACAATTCTTGAGTAAAAGAA
HShell075	ACGACAAACAAATAAATCCTAATTTACCAACGCAATAATAAACCATAA
HShell076	TCGTTAGAAACAGGAGCGCCAGAA
HShell077	TTAAAGGGATTCAAAGGGCGTGAACCATCAGGTTG
HShell078	GCGACTCGAATTCAATTCCACTAGTATTGGGCGCC
HShell079	ACACCCTGAGTCGCTATTAGCGATAGCTTAGATTCAA
HShell080	CCCTCAAATGACGACGACAGTACAATATTTTTGCGAA
HShell081	AGCCTCAGAGCATAAAGAATAAATCGGCTGACCACTG
HShell082	AGAAGACAATAAGAGGCGGTCATTAAAAATACCGAAAATGGCTATAGG
HShell083	CACCCTCATGAGACTCGGATTAGCCGTC
HShell084	ACCCACTTTGAACGGAACGAGGGCGCGACCTGTTT
HShell085	TTTTATACACTATAATGCCAAGTACGGTGGATGGC
HShell086	TGACCTGAGTTTCAGGGATAGCAAGCCCCTCA
HShell087	AATAATAAGATAAGCTCAACATGTTTTAA
HShell088	AAACAGACGTTGTAGGAATCATTACTTAGAGTCTG
HShell089	GCACGAATCTTAAGATTAGTAGCGACAGCGTCACC
HShell090	AATATCATTTCAATTACCTGAAACAAAATAGCAGCCCGCA
HShell091	GCGCAAATCAAGTGCCGTAAAGCACTAATAAAGGGAGAAAGGAGTGGC
HShell092	GCTGTTTTCATTTTTTGTTTAACGAAGCGAAT
HShell093	CCACTTTTGCTATAGAAAGGAACAACTAAAGG
HShell094	TTTGCTGGTCCAGTTTATTAAAGAACGTGGACTCCAACGTGCCC
HShell095	TTTATTTTACATTCAAAAGTCCTGGAGC
HShell096	TGACCGTAACAGAAGATAAAATGAAAAATTTT
HShell097	ACTGCGGATGTTTAGAGCTCCAAAACGTTGAA
HShell098	ACAACATTAAATTTTTGAAGGTTATCTATTTA
HShell099	TATTCAAAGACAAAAGGGCGGTCACAATATGC
HShell100	CAGCAGCAAACAGAGGTACAACATGGAGA
HShell101	GATCGGCATTTCTTTTCATAATCAAAATCCCT
HShell102	AGACGACGACACTATCAGTGAGAAAACAACTT

HShell103	CTTGCTTCAATCAATACAGGGAAGTTTACAGATTTG
HShell104	GGCGAGAACCATTGCATTGCCTGAAAATGGATTTCA
HShell105	AAGACTTCAAATATCGCCAAAGCGA
HShell106	CAGTTTTTGATGATACAGGAATAAAAGAAGTA
HShell107	TGTCGTGCCAGCTGCATGCGCGGGGTAAAGTGTAATT
HShell108	AGCCCGGAACCGCCACTACTTAGC
HShell109	ACTACCTTTGTTGGGTTACAATGAATACCGAAGTCAG
HShell110	TGGAAATAGAATAAACCTAGAAAAAGAACTGGTATT
HShell111	ACGCAACGCAAACAGACGACCGCGCCTGTGCCACGCATCAAACC
HShell112	CCAGAATTGAGAACGCCAACGACAAAAGAACATCGCCAGTATTA
HShell113	TACCGCCAGAGGAAGGGGGCAAGTGTAGCGGTCCCAC
HShell114	GCTAAAGCATCACCAGGAATTGTACATAACTAATA
HShell115	CGCTTATTATTCTTTTAACGGGGTCAGTTTAATTG
HShell116	ATGTTCAGTTGGACTAACAA
HShell117	GGTACCACCACCCCCTCAGA
HShell118	GAGAATTTTAGTGCCGAACAACCGAGGAGTTC
HShell119	AAATCAACCATCACATTTATCAGCTTGCAAATATT
HShell120	GTACGTCAATAGGTATTAAA
HShell121	CGCAAGGATAAAAATTTAATACTTCATAGGCTATACAGGCAGTA
HShell122	CTGCCTATTTAAGAGGCGCAGCGAACTTTTTCAACCA
HShell123	ATATAGCAAACGCTCAACAGTAGTATCACAGGCTC
HShell124	GCTACAATAGAAGGCAACATGTGTACTGATACGGA
HShell125	AGCCACCAAATGCAATGCCTGAGTAATGACCA
HShell126	CAGACGGGAGAATTAATTTCTTTTTTAATTTAACA
HShell127	ATGCTGATAATATAATGCTGTGGTCTGAGAG
HShell128	AGGGTGGTTAGACGGGCTGGTCATAGTCGACTCAGTGCCAA
HShell129	AATCACAAAGTCAACCCACAGAGGCAGGCCCT
HShell130	GAAGATCTACGTCATAACAAGAGAATCGATGAACGGGAAAAGCCCC
HShell131	TAGCCCAGAATGGCATTGACGAACCACCAGACTGA
HShell132	ATTTTGTTAATATTTATCATTATA
HShell133	GGATTCGCTTTTACATGTAGATTTTCAGGTTTATTT
HShell134	ATATAGAATCCTCGAAAGAGTACAACGGACCA
HShell135	CAAAATTAACGTCAGTGCCAGTTTTTG
HShell136	CCCCGGTTGATAATCATAATCGTACCAGTCAGGTGA
HShell137	TGCTGAGTTAAGTTGTGGGAAGGCAGTATAACGAATCC

HShell138	CAGAGGCAATAAAAGGGACAGTGAGGCCGAACGGTAGCCGA
HShell139	ATTACGATCTAAGCCTGTAGCATTCCACCCGT
HShell140	GGAAGATTGTATAAGCAAAAATTCGCATTATTAAATA
HShell141	TATCCAAGTTACAAAATCGCTAAATCGGTTGTAAT
HShell142	ATTTTTTAACTGGCCTTTAAGAGCAATAAAAACATTT
HShell143	TTCATTAAATAACCTACATTTTGACGGTACAATAT
HShell144	ACCATCAACAACCGCGCCCAATAGTGACGTTCTAG
HShell145	ATTTCATTCGCAGAGGGGAACCGATTCCCAGAGCC
HShell146	ATGGTGGTTAACCCATCGGCCGTCACCGCCGGCG
HShell147	CTCACGTAATCAAACAGCTGGCAGCAAGATCCTGTTTG
HShell148	CGGAGACAGTCAAATCTGTAGGTAACAAAGCTTCTTGACACGCC
HShell149	TGACTATTAATGTGCTGCGACGGCCTAGAGGATCC
HShell150	GTATTCGGAACCGAGGCTTGAATGACAATCTACCC
HShell151	CTGATAAATTAATATTGCCTGTAATCATTGACGTTGGCGGA
HShell152	AGGGGCTTTAAACGGAAACCTGCGGGCCTCCG
HShell153	TTTCAGGAGCCTGCCTTGAGGCGCCGACCAGG
HShell154	TGTTAAAGTACCATGTAATTTTAGTCTTTGAAAGC
HShell155	TCTGCGAACGCAACTAACTACGAAGATTAGAGAGGAA
HShell156	GCTTACGAGCACAAAGAGTCTGTCCAACCGCCATTCAGATCGG
HShell157	GATAATCACCGTCCAGGCGGATAAGTGCGGGG
HShell158	CCACAGTAATAAAAGAAGTTATAAAGGTAATTCAT

Table S2. Sequences of probe DNA and probe receptors strands which are tethered to the DNA nanoshell pore. The sequence segments of probe strand and receptors positioned within the pore lumen are in bold. To be tethered into the pore lumen, receptor strands DP-0nm, DP-3nm, and DP-6nm were added during the assembly to replace staples HShell118, HShell089, and HShell101, respectively.

ID	Sequence 5′ → 3′	Replaces
Probe DNA	**ACCTTCCTCCGCAATACTCC**	N/A
DP-0nm	GAGAATTTTAGTGCCGAACAACCGAGGAGTTC ttt **GGAGTATTGCGGAGGAAGGT**	HShell118
DP-3nm	GCACGAATCTTAAGATTAGTAGCGACAGCGTCACC ttt **GGAGTATTGCGGAGGAAGGT**	HShell089
DP-6nm	GATCGGCATTTCTTTTCATAATCAAAATCCCT ttt **GGAGTATTGCGGAGGAAGGT**	HShell101

Table S3. Sequences of biotin-modified DNA staple strands used for the assembly of the DNA nanoshell pore.

ID	Sequence 5′ → 3′
HSbiotin01	AAAATACGAAACACTCATCTTTGA ttt ATCTGCGAGCGACTA
HSbiotin02	AAACTAGCATGTCAAT ttt ATCTGCGAGCGACTA
HSbiotin03	GATTTAGGCAGGTAGAAAGATTCA ttt ATCTGCGAGCGACTA
HSbiotin04	CGTGGGAACGAGTAACAACCCGTC ttt ATCTGCGAGCGACTA
HSbiotin05	TCTGCCAGAGATGGGCGCATCGTA ttt ATCTGCGAGCGACTA
HSbiotin06	ATTTTTGCGTCTGGAAGTTTCATT ttt ATCTGCGAGCGACTA
HSbiotin07	AACAGAGAAACACCAGAACG ttt ATCTGCGAGCGACTA
HSbiotin08	TAAATCAATGAATTTCTTAAACAG ttt ATCTGCGAGCGACTA
HSbiotin09	CAGTTGATACCATTAGATACATTT ttt ATCTGCGAGCGACTA
HSbiotin10	GAACAGAACCGGATATTCAT ttt ATCTGCGAGCGACTA
HSbiotin11	ATTCGCGTCCAATAGGAACGCCAT ttt ATCTGCGAGCGACTA
HSbiotin12	TAGCGTAACCTTATGCGATTTTAA ttt ATCTGCGAGCGACTA
HSbiotin13	TCAACGTAAAGATTCAAAAGGGTG ttt ATCTGCGAGCGACTA
HSbiotin14	GACCGTACCTTTAATTGCTCCTTTTGAT ttt ATCTGCGAGCGACTA
HSbiotin15	GTCAATAAGCTGAAAAGGTGGCAT ttt ATCTGCGAGCGACTA
HSbiotin16	CTAATAGTAAGGCAAAGAATTAGC ttt ATCTGCGAGCGACTA
HSbiotin17	GAGAGATTATACCGAAATCCGCGACCTG ttt ATCTGCGAGCGACTA
HSbiotin18	ATTGGGCTGGGTAGCTATTTTTGA ttt ATCTGCGAGCGACTA

HSbiotin19	CAGTTCAGAAAACGAG ttt ATCTGCGAGCGACTA
HSbiotin20	AAAGTAAAGGCCGCTTTTGC ttt ATCTGCGAGCGACTA
HSbiotin21	TGAGGAAGTTTCCATT ttt ATCTGCGAGCGACTA
HSbiotin22	AGAGGACAGATGAACG ttt ATCTGCGAGCGACTA

Table S4. Sequences of DNA oligonucleotides used for control experiments. The blocker strand can bind to the probe strand attached to the AFM tip while the deblocker strand binds to the toehold region of the Blocker strand to facilitate strand displacement.

ID	Sequence 5′ → 3′
Blocker strand	TCG AAC ATG GTC TTG GTA GTA TTG CTG AGA AAG GT
Deblocker strand	CAG CAA TAC TAC CAA GAC CAT GTT CGA

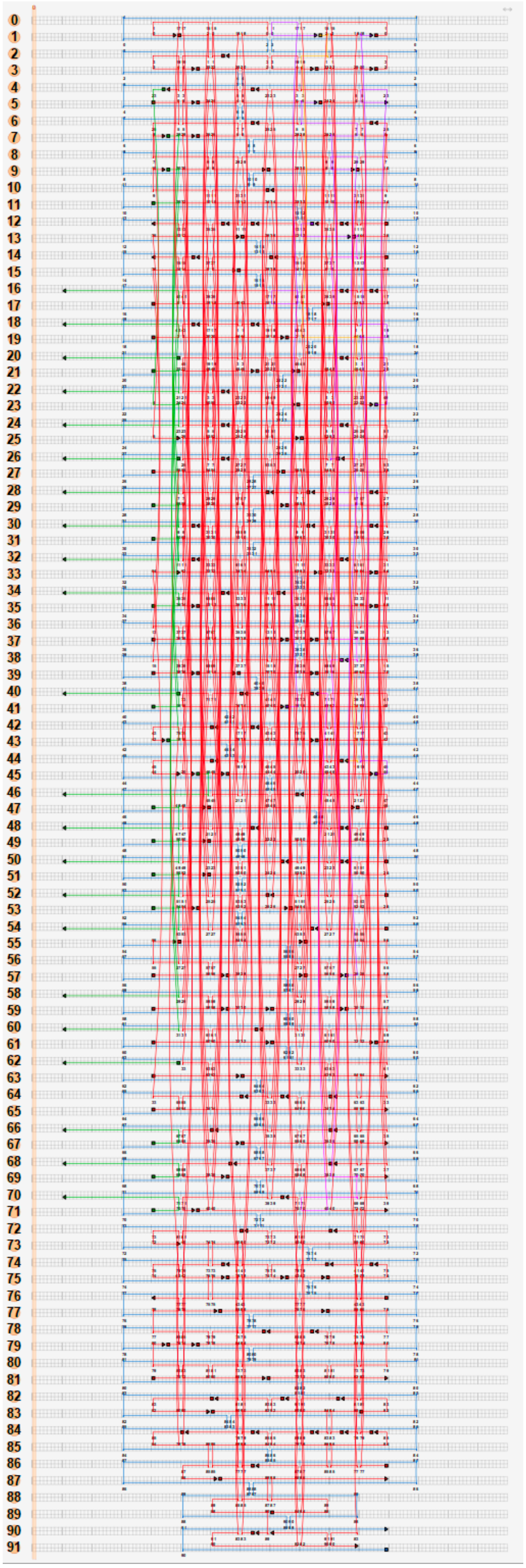

Figure S1. Connectivity map of the DNA nanoshell pore. Blue lines indicate the scaffold (m13mp18), red the staple strands, and green the single-stranded overhangs to which the biotin-modified strands bind.

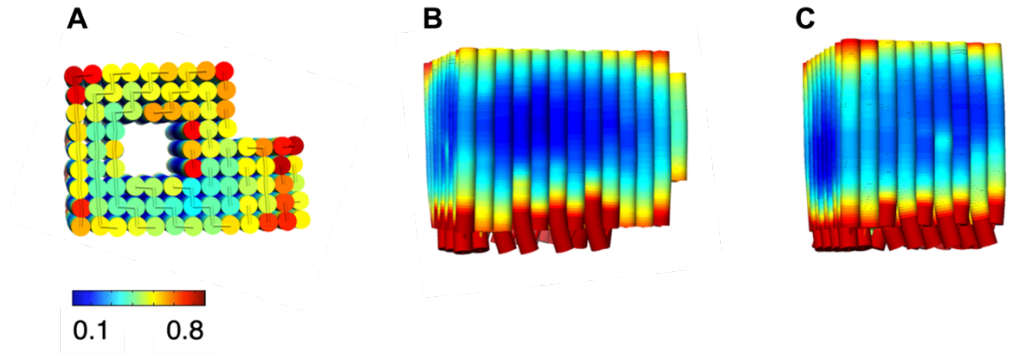

Figure S2. Thermal fluctuation analysis of the DNA nanoshell pore in A) Top, B) front, and C) side view. The analysis outlines high-stability regions (highlighted in blue) and lower stability regions towards the edges. Red rods at the pore bottom of high flexibility represent the single-stranded overhangs to which biotin-labeled strands bind. The analysis shows a minimal RMSF (root mean square fluctuation) of 0.1 nm and a maximum one of 0.8 nm. The analysis was carried out via the CanDo online server.[1,2]

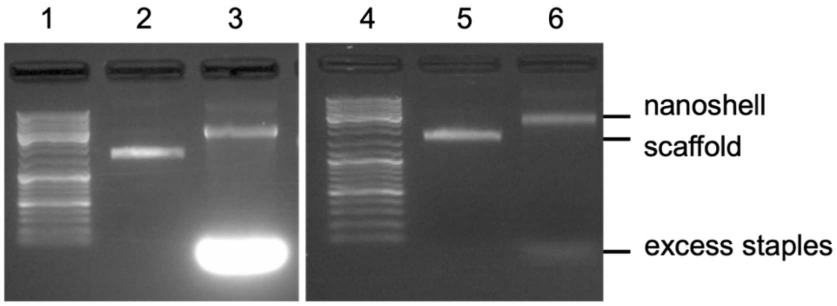

Figure S3. 1% Agarose gel electrophoresis analysis on the formation of the DNA nanoshell pore. Lanes from left to right: (1) 1 kb ladder, (2) M13mp18 scaffold, (3) assembled nanoshell pore, (4) 1kb ladder, (5) M13mp18 scaffold, (6) assembled nanoshell pore after gel purification and removal of excess staple strands.

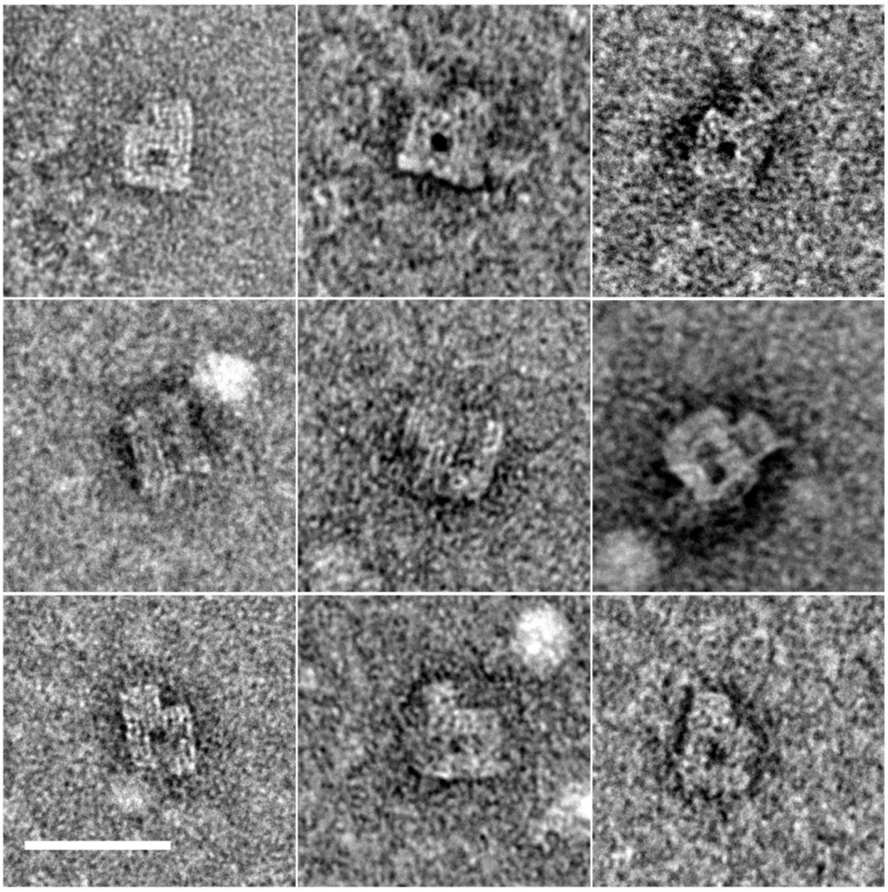

Figure S4. Representative TEM images of negatively stained DNA nanoshell pores. Scalebar = 50 nm.

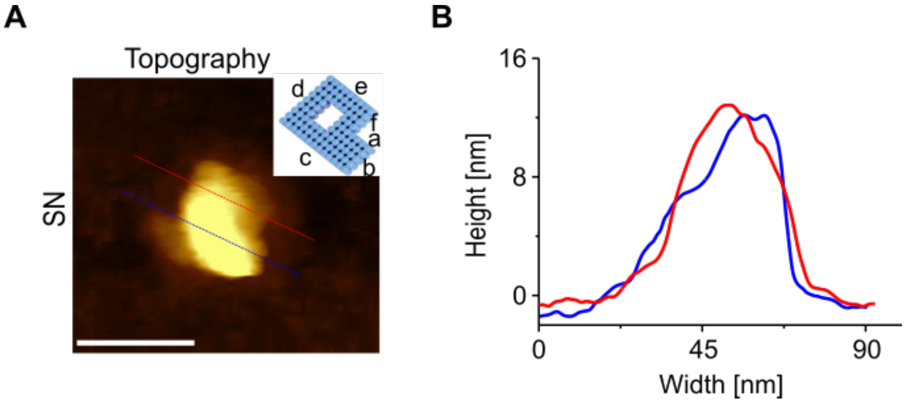

Figure S5. AFM analysis of the geometry of the DNA nanoshell pore. A) AFM topography of a pore on a streptavidin crystal layer and schematic design of the pore in top view (inset). The dimensions a-f corresponds to the data in Table S5. Scalebar = 50 nm. B) Height profiles of the pore along lines in the AFM topography image.

Table S5. AFM and TEM-derived dimensions of the DNA nanoshell pore.

Pore indications	Pore section	Dimension [nm]	n
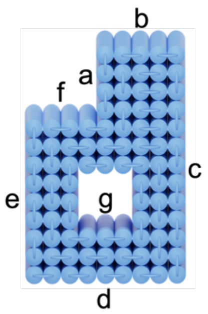	**From AFM**		
	a	13.4 ± 1.7	10
	b	15.2 ± 1.9	10
	c	39.7 ± 2.1	10
	d	30.3 ± 2.6	10
	e	29.1 ± 2.8	10
	f	14.7 ± 1.5	10
	height	13.6 ± 2.2	10
	**From TEM**		
	g	7.1 ± 0.4	21
	c	31.7 ± 1.3	15
	d	22.4 ± 0.9	16
	height	26.7 ± 2.6	9

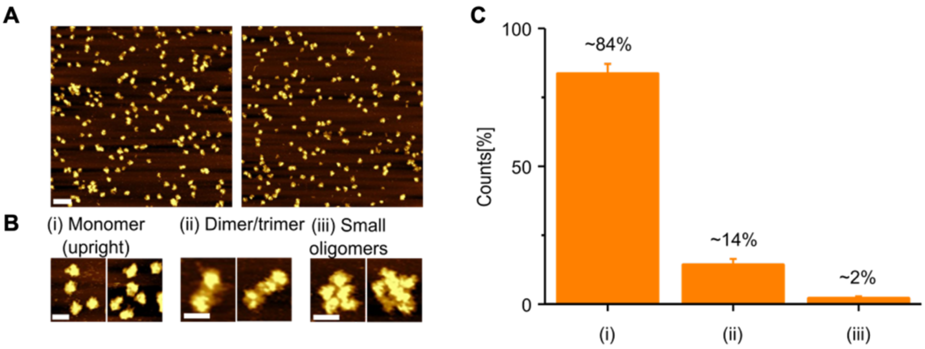

Figure S6. Binding orientation of DNA nanoshell pores on 2D streptavidin crystal layer, as analyzed by AFM. A) Representative images of pores adsorbed onto streptavidin-decorated mica plates imaged in tapping mode. Horizontal scale, 300 nm. B) High-resolution scan showing (i) single pores in upright position on streptavidin, (ii) dimers and trimers, and (iii) clusters of small oligomers. Horizontal scale bar, 50 nm. C) Distribution of monomers, dimers and trimers, and cluster (n = 329).

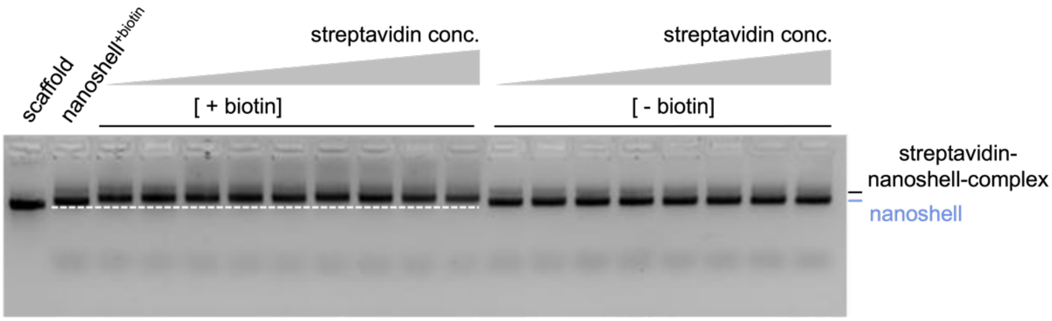

Figure S7. Gel electrophoretic mobility shift analysis on the binding of biotin-modified DNA nanoshell pores to streptavidin. 1% Agarose gel (run for 90 min at 65 V) displaying scaffold (lane 1), assembled nanoshell$^{+biotin}$ (lane 2; 10 µL, 5 nM) incubated with streptavidin (lanes 2-11, 0-20 µL of 15 µM), non-modified nanoshell pore incubated with streptavidin (lanes 12-19, 0-20 µL, 15 µM). Upshifted gel bands (lanes 3-11) indicate streptavidin binding to biotin-modified nanoshell pores compared to a no shift for the unmodified structure.

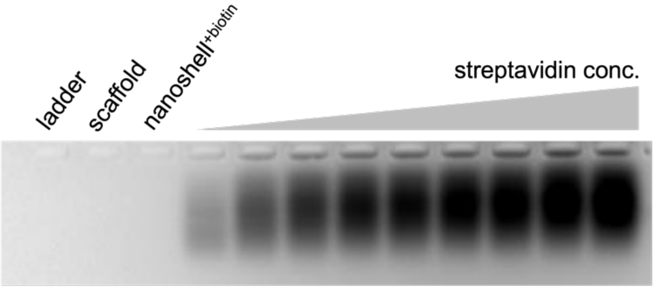

Figure S8. Gel electrophoretic mobility shift analysis on the binding of fluorescently labeled streptavidin to the biotinylated DNA nanoshell pore. 1% Agarose gel (run for 90 min at 65 V) on the M13mp18 scaffold, purified biotinylated DNA nanoshell pore (10 µL, 5 nM) and (lanes 3-11) purified biotinylated nanoshell pore (10 µL, 5 nM) incubated with Alexa 647-labeled streptavidin at increasing concentrations (0-20 µL, 15 µM). The bands were visualized to show the Alexa 647 streptavidin binding.

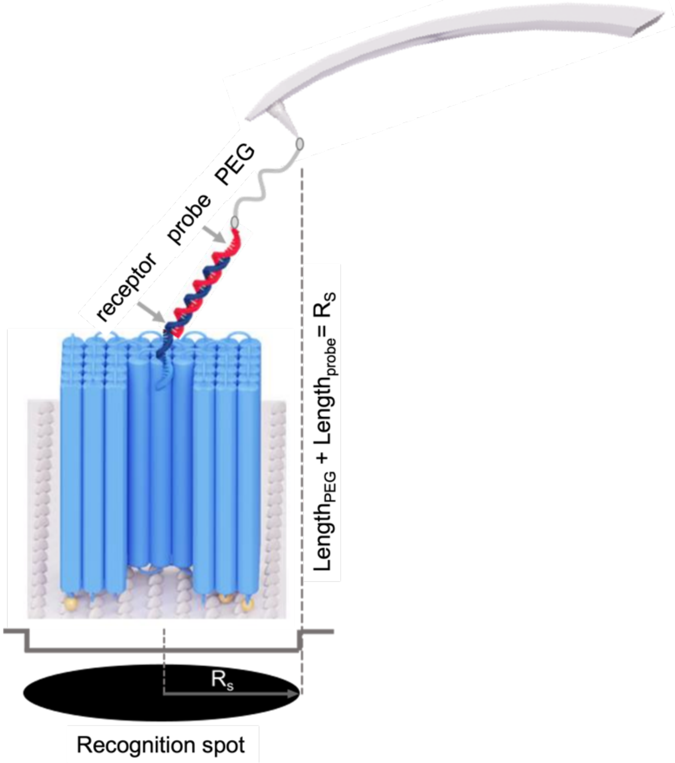

Figure S9. Schematic illustration on how the size of recognition spots in TREC analysis depends on the position of the receptor binding sites within the DNA nanoshell pores, as illustrated for the DP-0nm pore. Rs was determined from the lengths of the PEG linker, probe DNA, short TTT spacer anchoring of the receptor DNA to the pore wall, and an amino modifier linking the probe DNA to the AFM tip.

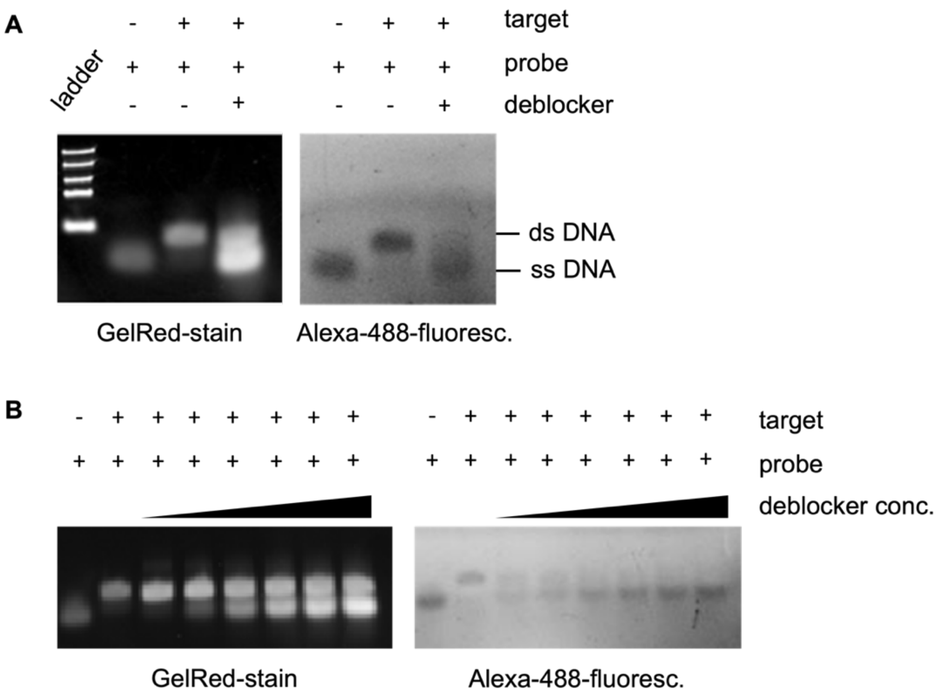

Figure S10. PAGE mobility shift analysis on the binding of the blocker to the probe strands, and its toehold-mediated removal by the deblocker strand. A) 10% Native PAGE displaying 100 bp ladder, probe DNA, receptor-probe DNA duplex, and the duplex after addition of deblocker strand to replace the probe strand from the duplex. As the probe strand was fluorophore-labeled, its tunable presence in the duplex was tracked in the fluorescence-based images of the gel. B) PAGE analysis on titrating the probe-blocker strand against increasing concentrations of deblocker DNA.

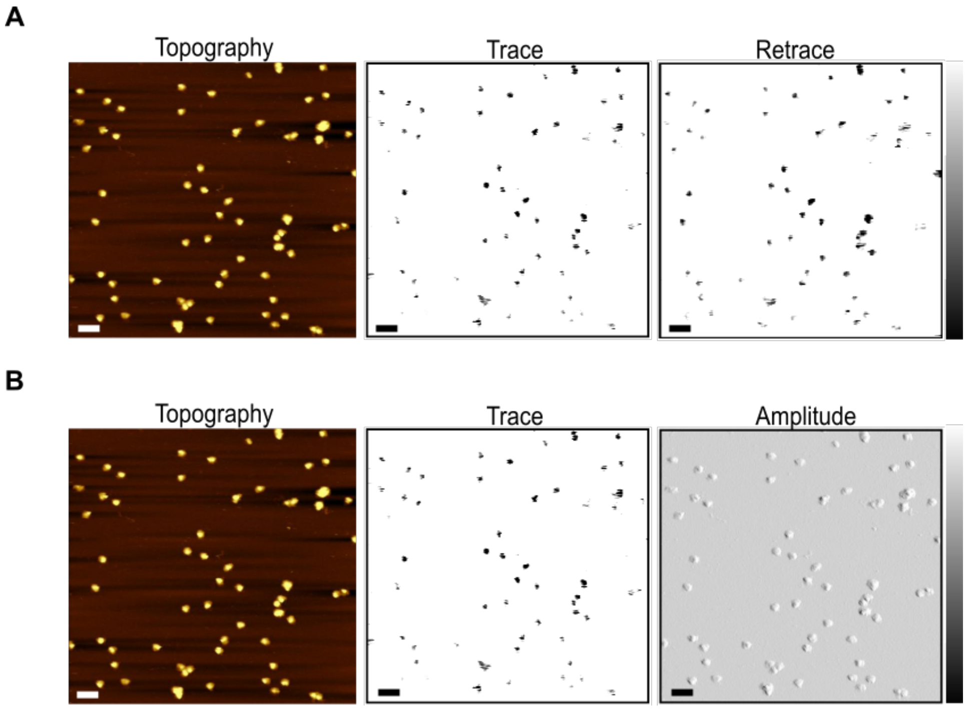

Figure S11. Verification of recognition signals. A) The recognition spots were verified by comparing the recognition signals obtained from the trace (middle panel) and retrace (right panel) images of cargo-receptor binding. B) Recognition spots verified by comparing the recognition signals obtained from the trace (middle panel) and amplitude (error) image (right panel) of cargo-receptor binding. Recognition spots (dark spots) do not change from trace to retrace images. In addition, no distinct dark spots are visible in the amplitude image. The latter two findings unequivocally confirm the specificity of the recognition signals. Horizontal scale: 200 nm, vertical scale of recognition and amplitude: 0.1 V and 0.3 V respectively.

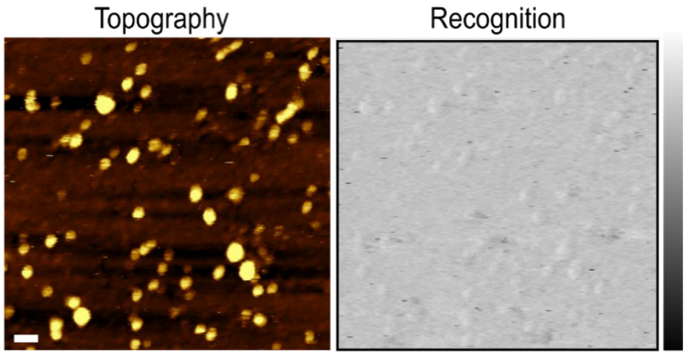

Figure S12. Control experiments using non-functionalised AFM tips. No visible recognition signals were observed when the shell nanopores were imaged with non-functionalised tips or tips bearing no cargo DNA. Left panels are topography images of DNA nanoshells on streptavidin layer. The right panels are the corresponding recognition images. Horizontal scale: 200 nm, Vertical scale of recognition is 0.35 V

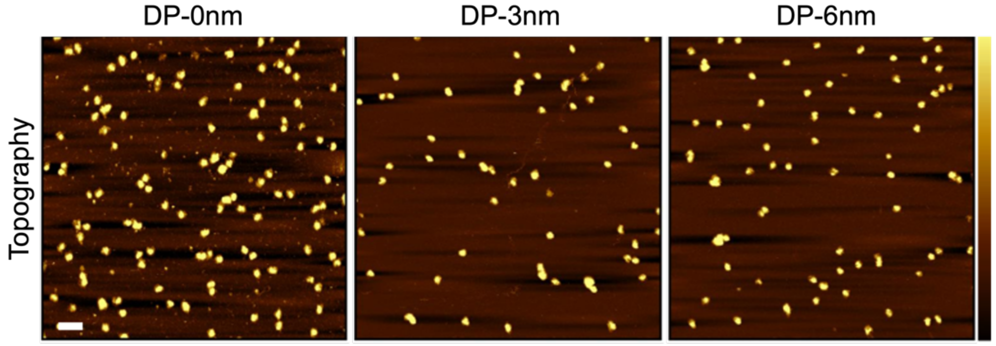

Figure S13. AFM topography images of DNA nanoshell pores DP-0nm, DP-3nm, and DP-6 nm within TREC imaging. All measurements were performed using tapping mode in liquid.

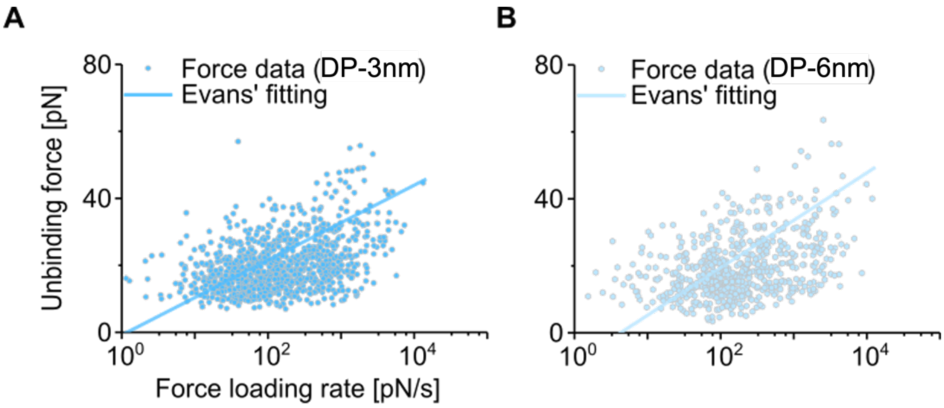

Figure S14. Dynamic force spectra plots on the interaction of tip-tethered probe DNA with receptor DNA tethered to the DNA nanoshell pores. The plots display the dependence of unbinding force on the force loading rate with A) the receptor at 3 nm depth and B) the receptor at 6 nm depth. Evans fitting represent a single energy barrier of the probe–receptor DNA bond; n=3; population data cut at μ ± 2σ.

3. Note A

TREC analysis of probe–receptor DNA binding

The Gwyddion software image analysis program was used to analyze the simultaneously acquired topography and recognition images. For image processing of topography images, two filtering parameters X and Y were applied to select for fully formed nanoshell pores after flattening with first-order polynomial function in Gwyddion.

$$Filter = X \cup Y$$

X is the nominal radius (X ≥ 11.25 nm) and Y is the projected area above the streptavidin layer (Y ≥ 631.25 nm^2, which corresponds to the nominal surface area of the nanoshell pore).

Using the above criterion, any protrusions above the streptavidin layer were selected by the filter using the masking function in Gwyddion. Each singular masked object was then analyzed to determine its maximum height, and mean radius in Gwyddion. The data was processed in Origin to determine mean values and standard deviation and plotted as histograms.

For image processing of recognition images, all recognition events were counted when the DC voltage signal level decreased below the thermal noise level on the background signal.[34] The recognition threshold was set from the reduction of the amplitude in the upper parts of the signal by at least 3 folds of the root mean square value.[5]

$$Recognition_{threshold} \leq A - 3 * RMS$$

RMS was determined by first of all selecting at least 5 different regions on leveled recognition images having no recognition or topography cross-talk. From this, the RMS values were extracted using the statistics tool in Gwyddion and from these the mean values were computed.

Furthermore, for each individual DNA nanopore construct, a separate filtering criterion was applied to analyze its recognition images as illustrated below:

For DP-0nm with receptors at 0 nm,

$$Filter = X \geq 18\ nm$$

$$L_{PEG} + L_{target} + L_{ttt} + L_a = X$$

$$\sim 9\ nm + \sim 7\ nm + \sim 1\ nm + \sim 1\ nm = 18\ nm$$

For DP-3nm with receptors at 3 nm,

$$Filter = X \geq 15\ nm$$

$$L_{PEG} + L_{target} + L_{ttt} + L_a = X - 3$$

$$\sim 9\ nm + \sim 7\ nm + \sim 1\ nm + \sim 1\ nm = 18\ nm - 3\ nm = 15\ nm$$

For DP-6nm with receptors at 6 nm,

$$Filter = X \geq 12\ nm$$

$$L_{PEG} + L_{target} + L_{ttt} + L_a = X - 6\ nm$$

$$\sim 9\ nm + \sim 7\ nm + \sim 1\ nm + \sim 1\ nm = 18\ nm - 6\ nm = 12\ nm$$

X… radius of recognition spot,

ttt … short thymine base spacer linking probe receptor to the pore wall; a: amino modifier spacer

4. Note B

PDF analysis of unbinding forces

The probability density function (PDF) of the unbinding force was constructed[6] from every unbinding event on the same nanopore construct at the same pulling velocity. For each unbinding force value, a Gaussian of unitary area with its center representing the unbinding force and the width (standard deviation) reflecting its measuring uncertainty (square root of the variance of the noise in the force curve) was computed.

All Gaussians from one experimental setting were accordingly summed up and normalized with its binding activity to yield the experimental PDF of unbinding force. The maximum of the PDF reflects the most probable unbinding force, F of the bond and can be easily extracted with Gaussian fitting according to a standard procedure in the literature.[7]

Importantly, the uncertainty of determining the most probable unbinding forces (standard error of the peak position from Gaussian fitting, is proportional to the square root of the sum of the squared residuals and inversely proportional to the square root of the number of data points (unbinding events), detailed formula in the report by Richter[8] is very low, which is less than 1 pN for all peaks in this manuscript.

For example, in Fig 5E, the most probable unbinding forces for DP-0nm, DP-3nm and DP-6nm were 24.18 ± 0.84 pN, 18.95 ± 0.85 pN, 12.37 ± 0.83 pN where the force values, F are the maximum position ± standard deviation. Standard deviation is sigma divided by the square root of the number of unbinding events (n = 110, n = 96, n = 52 for DP-0nm, DP-3nm, DP-6nm respectively per experiment).

Thus, the force measurements easily have the capability to clearly distinguish the unbinding forces arising from different sites for all data shown in this manuscript.

5. References

1. Castro, C. E. *et al.* A primer to scaffolded DNA origami. *Nat. Methods* **8**, 221–229 (2011).
2. Kim, D. N., Kilchherr, F., Dietz, H. & Bathe, M. Quantitative prediction of 3D solution shape and flexibility of nucleic acid nanostructures. *Nucleic Acids Res.* **40**, 2862–2868 (2012).
3. Lin, L., Wang, H., Liu, Y., Yan, H. & Lindsay, S. Recognition imaging with a DNA aptamer. *Biophys. J.* **90**, 4236–4238 (2006).
4. Shubham, S. *et al.* A 2′FY-RNA motif defines an aptamer for Ebolavirus secreted protein. *Sci. Rep.* **8**, 12373 (2018).
5. Stainer, S. *et al.* Single molecule distribution of RhD binding epitopes on ultraflat erythrocyte ghosts. *Nanoscale* **12**, 22097–22106 (2020).
6. Baumgartner, W., Hinterdorfer, P. & Schindler, H. Data analysis of interaction forces measured with the atomic force microscope. *Ultramicroscopy* **82**, 85–95 (2000).
7. Evans, E. Looking inside molecular bonds at biological interfaces with dynamic force spectroscopy. *Biophys. Chem.* **82**, 8397 (1999).
8. Richter, P. H. Estimating errors in least-squares fitting. *Telecommun. Data Acquis. Rep.* (1995).
